# Antagonism between neuropeptides and monoamines in a distributed circuit for pathogen avoidance

**DOI:** 10.1016/j.celrep.2024.114042

**Published:** 2024-04-03

**Authors:** Javier Marquina-Solis, Likui Feng, Elke Vandewyer, Isabel Beets, Josh Hawk, Daniel A. Colón-Ramos, Jingfang Yu, Bennett W. Fox, Frank C. Schroeder, Cornelia I. Bargmann

**Affiliations:** 1 Lulu and Anthony Wang Laboratory of Neural Circuits and Behavior, The Rockefeller University, New York, NY 10065, USA; 2 Department of Biology, KU Leuven, 3000 Leuven, Belgium; 3 Program in Cellular Neuroscience, Neurodegeneration and Repair, Departments of Neuroscience and of Cell Biology, Yale University School of Medicine, New Haven, CT 06511, USA; 4 Instituto de Neurobiología José del Castillo, Recinto de Ciencias Médicas, Universidad de Puerto Rico, San Juan, PR 00901, USA; 5 Wu Tsai Institute, Yale University, New Haven, CT 06510, USA; 6 Boyce Thompson Institute and Department of Chemistry and Chemical Biology, Cornell University, Ithaca, NY 14853, USA

**Keywords:** Caenorhabditis elegans, pseudomonas aeruginosa PA14, bacterial pathogens, pathogen response, sickness behavior, neuropeptide signaling, neuromodulatory circuits, neural circuits, behavior, Pathogen avoidance

## Abstract

Pathogenic infection elicits behaviors that promote recovery and survival of the host. After exposure to the pathogenic bacterium *Pseudomonas aeruginosa* PA14, the nematode *Caenorhabditis elegans* modifies its sensory preferences to avoid the pathogen. Here, we identify antagonistic neuromodulators that shape this acquired avoidance behavior. Using an unbiased cell-directed neuropeptide screen, we show that AVK neurons upregulate and release RF/RYamide FLP-1 neuropeptides during infection to drive pathogen avoidance. Manipulations that increase or decrease AVK activity accelerate or delay pathogen avoidance, respectively, implicating AVK in the dynamics of avoidance behavior. FLP-1 neuropeptides drive pathogen avoidance through the G protein-coupled receptor DMSR-7, as well as other receptors. DMSR-7 in turn acts in multiple neurons, including tyraminergic/octopaminergic neurons that receive convergent avoidance signals from the cytokine DAF-7/transforming growth factor β. Neuromodulators shape pathogen avoidance through multiple mechanisms and targets, in agreement with the distributed neuromodulatory connectome of *C. elegans*.

## Highlights

• *C. elegans* avoids the pathogen *P. aeruginosa* after prolonged exposure
• FLP-1 neuropeptides released by two AVK neurons drive pathogen avoidance
• FLP-1 neuropeptides act through the G protein-coupled receptor DMSR-7
• The peptidergic signaling pathway antagonizes tyraminergic/octopaminergic neurons

## Graphical abstract

**Figure:**
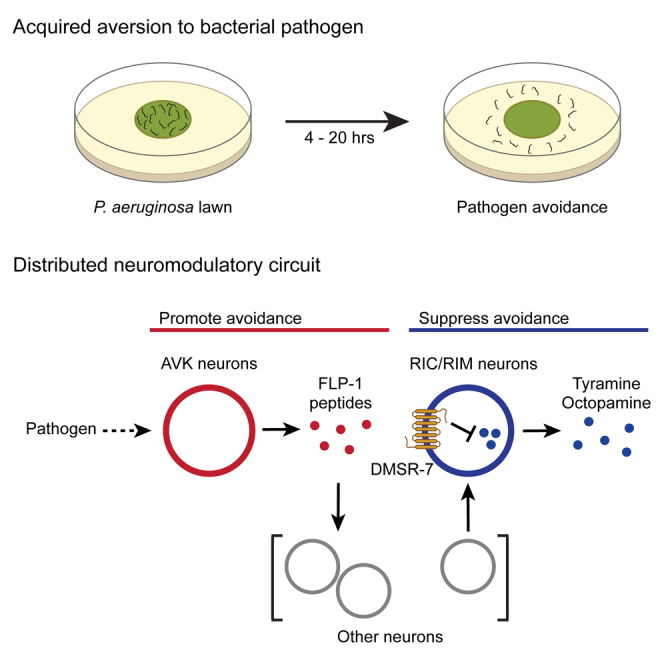

## Teaser

Marquina-Solis et al. show that RF/RYamide FLP-1 neuropeptides promote an acquired pathogen avoidance behavior in *C. elegans*. FLP-1 neuropeptides regulate multiple targets in a distributed neuromodulatory circuit, including tyraminergic/octopaminergic neurons that inhibit pathogen avoidance. Pathogen exposure increases expression and release of *flp-1* and decreases expression of a tyramine/octopamine biosynthetic gene.

## Introduction

Animals reorganize their behaviors during pathogenic infection to reduce energy expenditure and facilitate recovery.^1^^,^^2^^,^^3^^,^^4^^,^^5^ These behavioral modifications, collectively known as sickness behavior, include altered sensory preferences, lethargy, decreased feeding, and decreased sociability.^6^^,^^7^ The neural circuits and mechanisms used by the brain to sense infection and coordinate the resulting behavioral responses are areas of active investigation.^8^^,^^9^

The nematode *C. elegans* interacts with a variety of bacteria in its natural environment, including both nutritious and pathogenic *Pseudomonas* species.^10^^,^^11^^,^^12^^,^^13^ Among these, the opportunistic pathogenic bacteria *Pseudomonas aeruginosa* PA14 is a well-studied model of *C. elegans* pathogenesis that elicits innate immunity as well as behavioral defense responses.^14^^,^^15^^,^^16^^,^^17^ *C. elegans* is initially attracted to PA14 bacteria, but after several hours, the infected animals abandon the PA14 lawn and subsequently avoid *Pseudomonas* odors. Failure to avoid PA14 results in death after approximately 72 h of exposure, while PA14 avoidance prolongs survival.^18^ The transition from an attracted state to an avoidance state is also observed with other pathogens and toxic foods and represents a well-defined defensive behavior.^19^^,^^20^^,^^21^

Previous studies have identified several sensory cues that regulate acquired PA14 avoidance.^18^^,^^22^ The ASJ chemosensory neurons recognize two bacterial metabolites made by PA14, phenazine-1-carboxamide (PCN) and pyochelin, and in response they express and secrete the cytokine transforming growth factor β (TGF-β)/DAF-7.^22^ TGF-β signaling activates PA14 avoidance by suppressing animals’ preference for the low oxygen levels in a PA14 lawn.^22^ Conversely, *npr-1* mutants, which have enhanced low-oxygen preference, fail to avoid PA14.^18^ Reducing environmental oxygen restores PA14 avoidance to *daf-7* and *npr-1* mutants, suggesting that oxygen preferences interact with a second system that can recognize the pathogen and promote avoidance behavior.^22^

While the sensory cues PCN, pyochelin, and oxygen are present throughout PA14 exposure, PA14 avoidance develops after several hours through internal detection of the pathogen. Here, we describe a peptidergic signaling pathway that sets the tempo of the acquired avoidance behavior. Using genetic and molecular tools, we demonstrate that AVK interneurons drive PA14 avoidance by releasing NPF/NPY-like neuropeptides encoded by *flp-1*. FLP-1 neuropeptides act on multiple cells including tyraminergic and octopaminergic neurons that oppose pathogen avoidance. Signals from FLP-1 and the cytokine DAF-7 converge on a shared circuit that integrates internal and external signals to regulate aversive behavior.

## Results

### Neuropeptide processing affects pathogen avoidance

**Figure 1:**
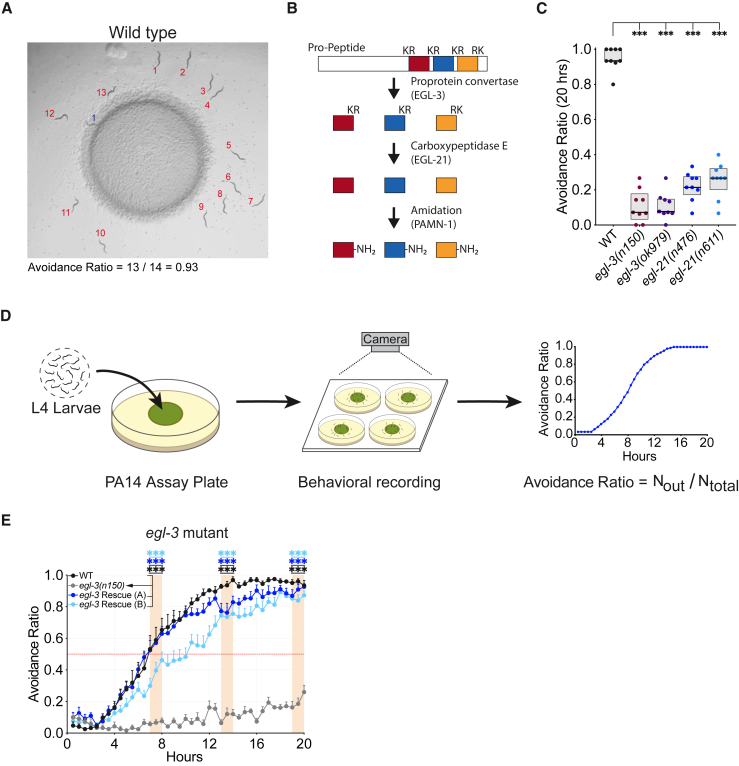
Neuropeptide-processing enzymes are required for PA14 avoidance (A) Wild-type (WT) animals after 20 h exposure to a *Pseudomonas aeruginosa* PA14 lawn. Red numbers indicate animals outside the lawn, and blue number indicates animal inside the lawn. (B) Schematic of neuropeptide processing. (C) Avoidance ratio of WT animals and *egl-3(n150)*, *egl-3(ok979)*, *egl-21(n476)*, and *egl-21(n611)* loss-of-function mutants calculated 20 h after exposure to PA14. (D) Schematic of dynamic PA14 avoidance assay. In all figures, a single assay represents the behavior of 11–17 animals on a single plate. (E) The PA14 avoidance defect of *egl-3(n150)* mutants can be rescued by expression of a genomic fragment containing the *egl-3* gene. Avoidance ratios at hours 7–8, 13–14, and 19–20 after PA14 exposure were compared for test animals and controls tested in parallel on the same day. For statistics, see Table S4. For (C), *n* = 9 assays for all groups; for (E), *n* = 8 assays for all groups. Graphs are mean + SEM. ^∗∗∗^p < 0.001, by one-way repeated-measures analysis of variance (ANOVA) with Dunnett’s post hoc test; (C) comparisons to WT, (E) comparisons to *egl-3(n150)*.

To identify molecular mechanisms that drive pathogen avoidance, we examined 67 mutants affecting neurotransmitters, neuropeptides, gap junctions, sensory function, innate immunity, and stress responses in a PA14 avoidance assay (Table S1). For the initial screen, we exposed L4 stage animals to a small lawn of PA14 bacteria and calculated the fraction of adult animals outside the lawn after 20 h (avoidance ratio) (Figure 1A). Over 90% of wild-type animals were outside the PA14 lawn after 20 h, but this behavior was greatly reduced in a subset of tested strains (Table S1). Among these were mutants in *egl-3,* which encodes proprotein convertase type 2, an enzyme that converts pro-peptides into neuropeptide intermediates (Figure 1B).^23^ Two *egl-3* loss-of-function mutants showed strong PA14 avoidance defects such that less than 15% of animals avoided PA14 at 20 h (Figure 1C). A second gene affecting neuropeptide processing, *egl-21*, encodes a carboxypeptidase E that releases C-terminal basic amino acids from neuropeptide precursors.^24^ Like *egl-3* mutants, *egl-21* loss-of-function mutants showed reduced PA14 avoidance (Figure 1C). These results suggest that PA14 avoidance is modulated by neuropeptides.

To gain insight into the dynamics of PA14 avoidance, individual assays were video recorded, and the avoidance ratio was calculated at 30 min intervals (Figure 1D). Wild-type animals started to avoid PA14 by 4 h after exposure, gradually increasing their avoidance until 95% of animals were outside the PA14 lawn (Figure 1E). To capture the gradual time course of the behavior, the avoidance ratio for each strain was compared to controls tested in parallel at 7–8, 13–14, and 19–20 h of exposure (see STAR Methods). *egl-3* mutants were defective in PA14 avoidance at all times, and two transgenic strains expressing an *egl-3* genomic fragment in the *egl-3(n150)* mutant background exhibited normal PA14 avoidance behavior at all times, confirming that the mutant phenotype was caused by loss of *egl-3* function (Figure 1E).

### The peptidergic AVK neurons mediate PA14 pathogen avoidance

**Figure 2:**
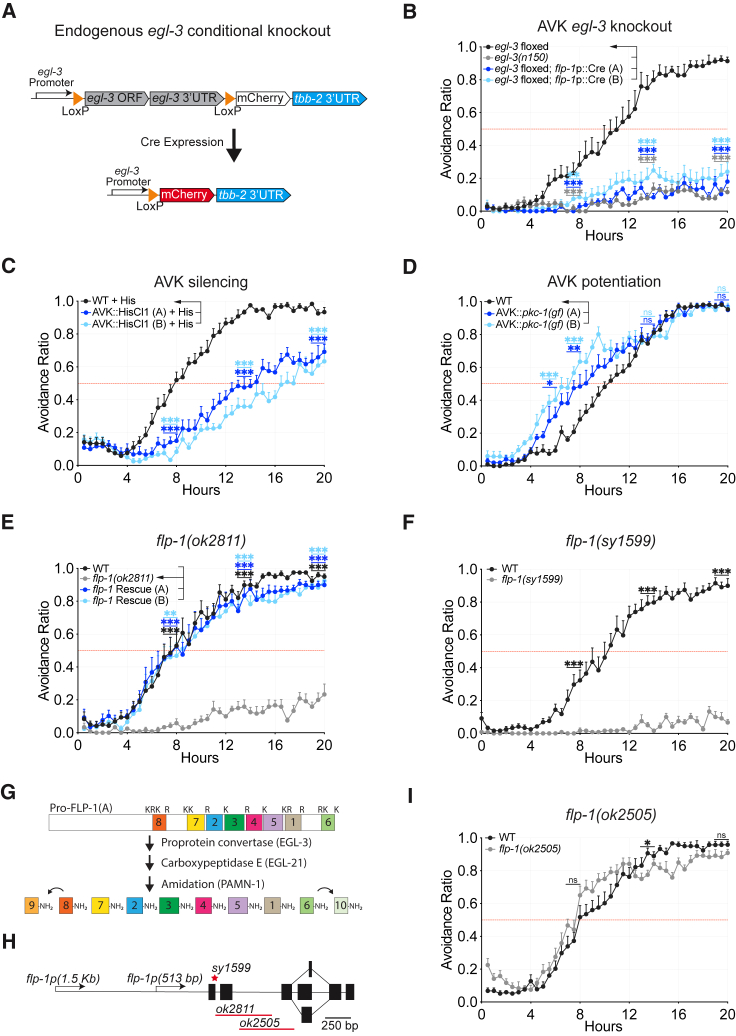
The AVK neurons promote PA14 avoidance via FLP-1 neuropeptides (A) Schematic of endogenous *egl-3* knockout strategy using Cre-lox recombination. In the *egl-3-*floxed strain, expression of Cre recombinase under cell-specific promoters excises the *egl-3* gene, allowing mCherry expression in the targeted cells. (B) Cell-specific knockout of *egl-3* using the *flp-1*(513 bp) promoter to drive Cre only in the AVK neurons; see also Figures S1E and S2E. (C) Silencing of the AVK neurons by cell-specific expression of the histamine-gated chloride channel HisCl1 delays PA14 avoidance; assays were supplemented with 10 mM histamine. (D) Potentiation of dense core vesicle release from the AVK neurons by cell-specific expression of a constitutively active protein kinase C (*pkc-1(gf)*) accelerates PA14 avoidance. (E) *flp-1(ok2811)* mutants are defective in PA14 avoidance and rescued by transgenic expression of a *flp-1* genomic fragment. (F) *flp-1(sy1599)* mutants are defective in PA14 avoidance. Additional data and controls are shown in Figure S2. (G) Schematic of the Pro-FLP-1-A precursor and the proteolytic pathway that yields active FLP-1 peptides. FLP-1-8 and FLP-1-9 are RYamide peptides, and all others are RFamides. FLP-1-9 and FLP-1-10 are derived from FLP-1-8 and FLP-1-6, respectively.^26^ (H) Schematic of the *flp-1* gene structure depicting 5′-regulatory regions, exons, splice isoforms, and mutations. There are two candidate null alleles, the *ok2811* deletion that disrupts the *flp-1* reading frame and the *sy1599* allele generated using CRISPR-Cas9 to insert an oligonucleotide with stop codons in all three reading frames in exon 1. The *ok2505* allele creates an in-frame deletion resulting in the loss of FLP-1 RYamide peptides KPNFMRYa and PNFMRYa (8 and 9 in G) and the FLP-1 RFamide peptide AGSDPNFLRFa (7 in G).^27^ (I) *flp-1(ok2505)* mutants lacking RYamide peptides have normal PA14 avoidance. For (B) and (D), *n* = 7 assays for all groups; for (C), (E), (F), and (I), *n* = 8 assays for all groups. Graphs are mean + SEM. ^∗^p < 0.05, ^∗∗^p < 0.01, and ^∗∗∗^p < 0.001, ns, not significant: (B–E) one-way ANOVA with Dunnett’s post hoc test and (F and I) unpaired two-tailed t test. Comparisons to (B) *egl-3* floxed, (C and D) WT, and (E) *flp-1(ok2811)*.

Neuropeptide genes can be expressed in all 302 neurons in the *C. elegans* nervous system, as well as the intestine and other non-neuronal cells.^25^ To identify cells that modulate PA14 avoidance, we generated a conditional *egl-3* allele by using CRISPR-Cas9 to insert LoxP sites flanking the endogenous *egl-3* locus (Figure 2A). Animals carrying the *egl-3*-floxed allele resembled wild-type animals in the PA14 avoidance assay (Figure S1B). Pan-neuronal expression of Cre recombinase to inactivate *egl-3* disrupted PA14 avoidance, while intestinal Cre expression did not affect the behavior (Figures S1C and S1D), pointing to a neuronal site of action. Next, we examined 25 transgenic lines expressing Cre under different neuronal promoters and quantified PA14 avoidance at 20 h (Figure S1A). Two strains with significant defects shared Cre expression in the AVK peptidergic neurons (Table S2). Targeted knockout of *egl-3* only in the AVK neurons, using two different cell-specific promoters, was sufficient to recapitulate the *egl-3* mutant defect (Figures 2B, S1A, and S1E). This result suggests that the *egl-3* defect can largely be explained by loss of neuropeptides from the AVK neurons, although we did not test all neurons, and others might play a role.

To ask if AVK acts acutely during PA14 avoidance, we expressed the *Drosophila* histamine-gated chloride channel HisCl1 in the AVK neurons. *C. elegans* does not appear to use histamine as a neurotransmitter but takes it up from the environment, allowing silencing of AVK::HisCl1 neurons by acute histamine addition.^28^ The time at which 50% of animals avoided PA14 was delayed by 5–8 h in animals in which AVK was silenced, and the avoidance plateau was reduced by ∼25% (Figure 2C). In the absence of histamine, PA14 avoidance by wild-type animals and AVK::HisCl1 animals was indistinguishable (Figure S2A). In a second experiment, we expressed the tetanus toxin light chain, which cleaves synaptobrevin protein to inhibit synaptic and peptidergic vesicle release, in AVK. AVK::tetanus toxin animals had a similar delay in PA14 avoidance to AVK::HisCl1 animals (Figure S2B). These experiments demonstrate that AVK activity and AVK vesicle release promote PA14 avoidance.

As a complementary experiment, we expressed a constitutively active protein kinase C (*pkc-1*(*gf*)) in the AVK neurons. *pkc-1* potentiates release of neuropeptidergic dense core vesicles and synaptic vesicles in *C. elegans*^29^^,^^30^; in the peptidergic AVK neurons, it is predicted to potentiate neuropeptide release. The time at which 50% of animals avoided PA14 was accelerated by ∼4 h in the AVK::*pkc-1*(*gf*) strain (Figure 2D). AVK::*pkc-1*(*gf*) animals did not avoid OP50, a non-pathogenic *E. coli* strain used as the standard laboratory food source (Figure S2C). Together, the inactivation experiments and gain-of-function experiments indicate that AVK neuropeptide secretion is rate limiting for PA14 avoidance.

### RF/RYamide FLP-1 signaling from AVK neurons increases upon pathogen exposure

Most *C. elegans* neurons express several neuropeptide genes.^25^ To further characterize the relationship between neuropeptide processing and AVK, we examined two neuropeptides that are highly enriched and expressed at high levels in AVK: *flp-1* and *nlp-49*.^25^^,^^31^ We found that *flp-1(ok2811)* and *flp-1(sy1599)* null mutants had a defect that closely resembled that of *egl-3* AVK-specific knockouts (Figures 2E and 2F), whereas *nlp-49* mutants were normal in PA14 avoidance (Figure S2D). The PA14 avoidance defect in *flp-1(ok2811)* was rescued by a *flp-1* genomic fragment that was expressed exclusively in AVK (Figures 2E and S2E). Although *flp-1* can be expressed in AIY as well as AVK neurons,^32^^,^^33^ the reporters and transgenes studied here appeared to be AVK specific (STAR Methods).

The *flp-1* gene encodes eight neuropeptides with a C-terminal RFamide sequence and two neuropeptides with a C-terminal RYamide sequence^26^^,^^34^^,^^35^ (Figure 2G; Table S3). These C-terminally modified peptides are related to NPF and NPY peptides in other species; we will refer to them collectively as FLP-1 neuropeptides.^36^ *flp-1(ok2505)* mutants, which lack FLP-1 RYamide peptides,^27^ showed normal PA14 avoidance, suggesting that the FLP-1 RFamide peptides are sufficient for avoidance behavior (Figures 2H and 2I). The *flp-1(ok2811)* allele was used for all subsequent studies.

**Figure 3:**
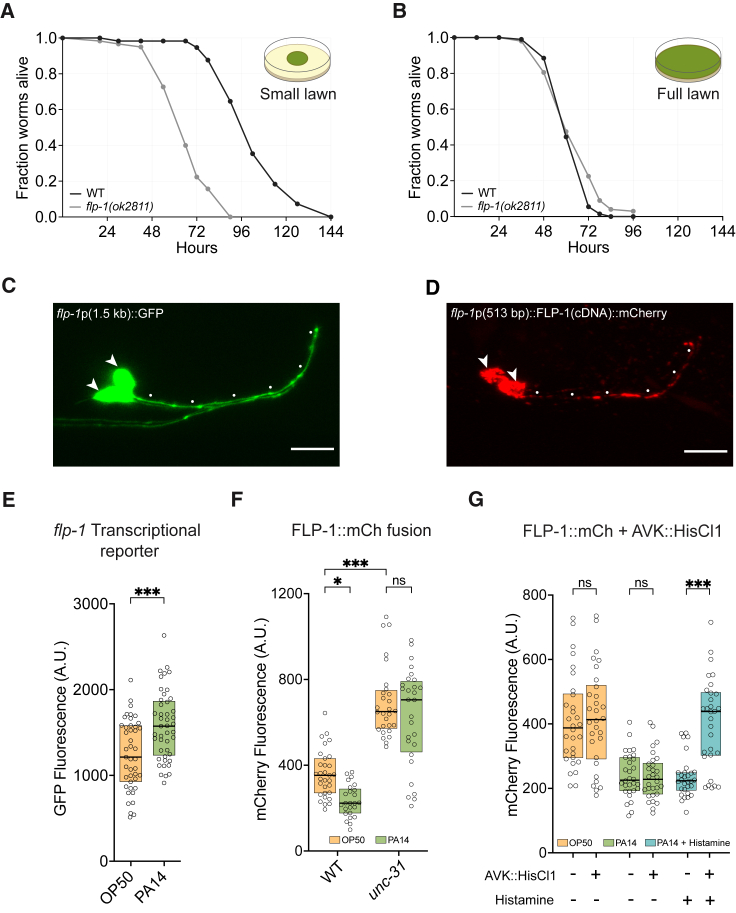
PA14 exposure modulates a reporter for FLP-1 neuropeptide release (A and B) Fraction alive over time for WT and *flp-1(ok2811)* mutants on small PA14 lawns (A) and full PA14 lawns (B). (C and D) Images showing AVK neurons expressing GFP under the *flp-1*(1.5 kb) promoter (C) and expressing FLP-1(cDNA)::mCherry under the *flp-1*(513 bp) promoter (D). Arrowheads indicate AVK cell bodies; dots indicate AVK axons. Anterior at right, dorsal up. Scale bars, 10 μm. (E) Animals expressing *flp-1*p(1.5 kb)::GFP exposed to *E. coli* OP50 or full PA14 lawns; exposure to PA14 upregulates the transcriptional reporter. (F) Animals expressing AVK::FLP-1(cDNA)::mCherry exposed to OP50 or full PA14 lawns. (Left) PA14 decreases FLP-1(cDNA)::mCherry levels in the AVK cell body. (Right) *unc-31(e928)* mutants have higher FLP-1(cDNA)::mCherry levels in the AVK cell bodies that are not decreased by PA14. The 513 bp promoter used to drive FLP-1::mCherry is not significantly induced by PA14 (Figure S2F). (G) Animals expressing AVK::FLP-1(cDNA)::mCherry and AVK::HisCl1 on OP50 lawns or full PA14 lawns with or without histamine. HisCl1/histamine blocks the PA14-dependent decrease in FLP-1(cDNA)::mCherry in AVK cell bodies. For (A) and (B), graphs are means from 3 (A) and 2 (B) independent experiments. For (E), *flp-1*p(1.5 kb)::GFP in OP50, *n* = 43, and in PA14, *n* = 45. For (F), WT *flp-1*p(513 bp)::FLP-1(cDNA)::mCherry in OP50, *n* = 31, and in PA14, *n* = 28; *unc-31(e928) flp-1*p(513 bp)::FLP-1(cDNA)::mCherry in OP50, *n* = 25, and in PA14, *n* = 28. For (G), all groups, *n* = 30. Boxplot center lines denote median; box ranges denote 25th–75th percentiles. ^∗^p < 0.05, and ^∗∗∗^p < 0.001, ns, not significant: (E) unpaired two-tailed t test, (F) two-way ANOVA with Tukey’s post hoc test, and (G) two-way ANOVA with Sidak’s correction.

To characterize the relationship between *flp-1* and pathogenesis, we compared the susceptibility of wild type and *flp-1* mutants to killing by PA14. On standard PA14 assay plates, *flp-1* mutants were more susceptible to PA14 killing than wild-type animals, with a t_50_ of killing at ∼60 h instead of ∼96 h (Figure 3A). To ask whether this susceptibility arises from reduced PA14 avoidance, killing rates were examined on plates uniformly seeded with PA14 so that neither *flp-1* mutants nor wild-type animals could avoid the pathogen. Under these conditions, both wild type and *flp-1* mutants had a t_50_ of killing at ∼60 h (Figure 3B). These results indicate that the increased susceptibility of *flp-1* mutants to killing by PA14 can be explained by their failure to avoid the bacterial pathogen.

To understand how pathogen exposure affects *flp-1* activity, we examined a transcriptional reporter in which a 1.5 kb fragment upstream of *flp-1* drove GFP expression (Figure 2H). This *flp-1* reporter gene was upregulated in AVK within 4 h of exposure to PA14, suggesting that PA14 exposure increases *flp-1* expression (Figures 3C and 3E).

Next, we examined a C-terminal FLP-1::mCherry fusion protein that has been used as a proxy for neuropeptide release from AVK^31^ (Figure 3D). AVK FLP-1::mCherry levels decrease in association with neuropeptide release and increase when release is blocked. Animals exposed to PA14 for 4 h had a ∼50% decrease in FLP-1::mCherry cell body fluorescence compared to animals in *E. coli* OP50, suggesting that PA14 increased FLP-1 release from AVK (Figure 3F). FLP-1::mCherry axonal fluorescence was not altered, in agreement with previous studies of this reporter.^31^ Recognizing that FLP-1::mCherry might detect processes other than neuropeptide release, we asked whether its properties were altered in mutants lacking *unc-31*/Ca^2+^-activated protein for secretion, which are deficient in neuropeptide secretion.^37^^,^^38^ *unc-31* mutants had increased basal FLP-1::mCherry fluorescence consistent with diminished neuropeptide release from AVK (Figure 3F). In addition, the PA14-induced reduction in FLP-1::mCherry fluorescence was absent in *unc-31* mutants as expected if that reduction correlates with neuropeptide release (Figure 3F). However, *unc-31* mutants are defective in neuropeptide release from all neurons throughout development, so the effect might be indirect. We therefore monitored FLP-1::mCherry after acutely and selectively silencing AVK neurons with HisCl1 and histamine to inhibit activity-dependent neuropeptide release. The AVK::HisCl1 transgene did not affect FLP-1::mCherry levels in the absence of histamine, but acute silencing during exposure to pathogen blocked the PA14-induced reduction in FLP-1::mCherry levels (Figure 3G). These results suggest that PA14 stimulates FLP-1::mCherry release through a process that requires AVK activity and dense core vesicle exocytosis.

### FLP-1 peptides signal through the G protein-coupled receptor (GPCR) DMSR-7 and other receptors

**Figure 4:**
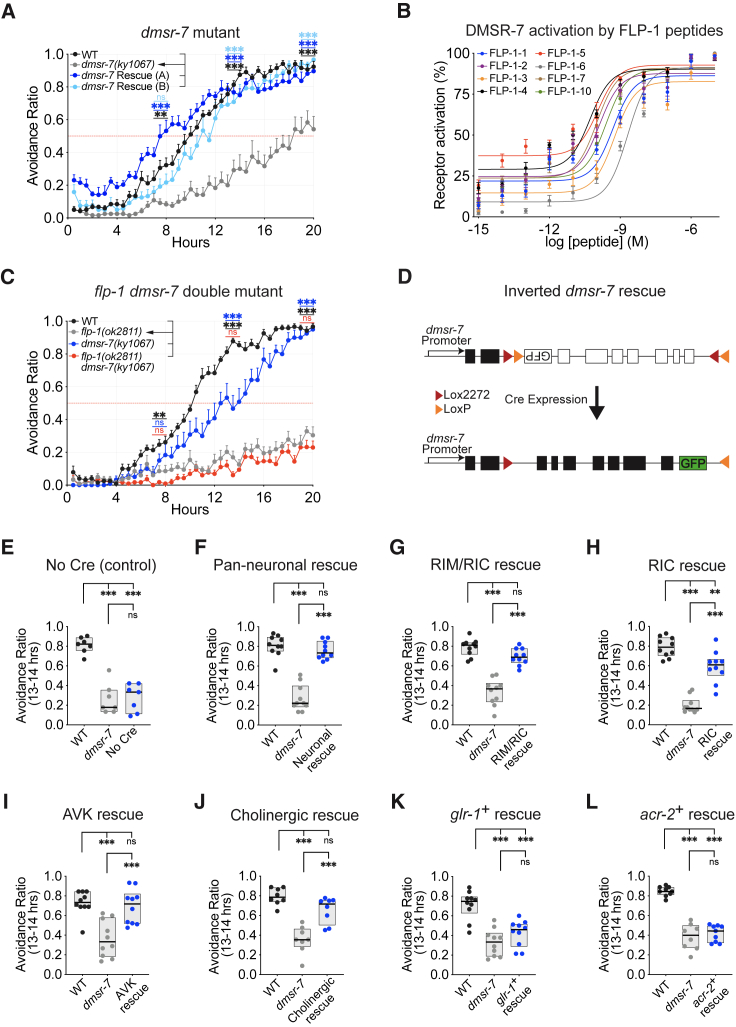
FLP-1 neuropeptides signal through the DMSR-7 receptor (A) Defective avoidance in *dmsr-7(ky1067)* mutants and rescue by transgenic expression of *dmsr-7* from its endogenous promoter. Additional receptor mutants are shown in Figure S3. (B) Aequorin bioluminescence (percentage of maximum receptor activation) of CHO cells expressing G_α16_ and DMSR-7 in response to different concentrations of FLP-1 peptides (#1–7, #10). For empty vector controls, see Figure S3J. (C) *dmsr-7* mutants are less defective in PA14 avoidance than *flp-1* mutants, and *flp-1 dmsr-7* double mutants resemble the more severe *flp-1* mutant. (D) Schematic of the transgene used to rescue *dmsr-7* in subsets of its endogenous expression pattern using the Cre-lox system. In all strains, cell-specific Cre-mediated reconstitution of the transgene was confirmed by GFP expression. (E–L) Avoidance ratios at 13–14 h on PA14. (E) Expression of the inverted *dmsr-7* transgene does not rescue *dmsr-7* avoidance behavior. (F–J) Rescue of *dmsr-7* avoidance behavior by Cre expression in all neurons (F), RIC and RIM interneurons (G), RIC interneurons (H), AVK interneurons (I), and a set of interneurons and motor neurons not including RIM, RIC, or AVK (J). (K and L) No rescue of *dmsr-7* avoidance behavior by Cre expression in RIM (and other interneurons) (K) or cholinergic motor neurons (L). Complete 20 h time courses for (E)–(L) are shown in Figure S4. For (A), (C), and (J), *n* = 8 assays for all groups; for (B), *n* = 6 assays for all groups; for (E), *n* = 7 assays for all groups; for (F)–(I) and (K), *n* = 10 assays for all groups; for (L), *n* = 8–9 assays for all groups. For (A) and (C), graphs are mean + SEM. For (E)–(L), boxplot center lines denote median; box ranges denote 25th–75th percentiles. ^∗^p < 0.05, ^∗∗^p < 0.01, and ^∗∗∗^p < 0.001, ns, not significant: (A and C) one-way ANOVA with Dunnett’s post hoc test, (A) comparisons to *dmsr-7(ky1067)*, (C) comparisons to *flp-1(ok2811)*, and (E–L) one-way ANOVA with Tukey’s post hoc test.

The *C. elegans* genome contains 158 neuropeptide precursor genes, encoding over 300 distinct neuropeptides, and more than 150 peptide GPCR genes.^39^ Previous screens of neuropeptide receptors using heterologous expression systems identified FRPR-7, NPR-4, NPR-5, NPR-6, and NPR-11 as receptors for FLP-1 neuropeptides.^31^^,^^36^ A recent comprehensive screen of *C. elegans* peptide GPCR candidates in CHO cells expressing the promiscuous G protein subunit G_α16_ identified additional FLP-1 receptors including DMSR-1, DMSR-5, DMSR-6, and DMSR-7.^39^ Examining loss-of-function alleles for each FLP-1 receptor candidate revealed that *dmsr-7(ky1067)* mutants were defective in PA14 avoidance, while *dmsr-1*, *dmsr-5*, *dmsr-6*, *npr-4*, *npr-5*, *npr-6*, *npr-11*, and *frpr-7* mutants were normal (Figures 4A and S3A–S3E; Table S1). CHO cells transfected with a *dmsr-7* cDNA generated Ca^2+^ responses upon exposure of FLP-1 RFamide peptides at concentrations of 0.05–3 nM (Figures 4B and S3J; Table S3), making DMSR-7 the highest-potency FLP-1 receptor we have identified.

PA14 avoidance in *dmsr-7* mutants was rescued by a transgene encompassing the *dmsr-7* genomic region, confirming that the defect was caused by loss of *dmsr-7* (Figure 4A). *dmsr-7* mutants were less defective in PA14 avoidance than *flp-1* mutants, and *flp-1 dmsr-7* double mutants resembled the more severe *flp-1* mutant, suggesting the involvement of additional receptors (Figure 4C). By testing double and triple receptor mutants (Figures S3F–S3I), we found that *dmsr-7 npr-6* double mutants were as defective as *flp-1* mutants in PA14 avoidance, although *npr-6* had no defects on its own (Figures S3F and S3D). Previous studies showed that NPR-6 and FRPR-7 in motor neurons redundantly regulate FLP-1-dependent body posture and locomotion,^31^ but PA14 avoidance in *npr-6 frpr-7* double mutants was normal (Figure S3G). These results point to DMSR-7 as an important receptor for FLP-1 in PA14 avoidance and NPR-6 as a potential second receptor.

The *dmsr-7* genomic fragment that rescued PA14 avoidance in *dmsr-7* mutants is expressed in motor neurons and interneurons but not in sensory neurons (Figure S3K).^25^ To identify neurons in which *dmsr-7* acts to induce PA14 avoidance, we used an inverted Cre-LoxP intersectional strategy to rescue *dmsr-7* in subsets of its endogenous expression pattern (Figure 4D). Control experiments showed that the inverted *dmsr-7* transgene did not rescue *dmsr-7* mutants, but PA14 avoidance was fully rescued upon pan-neuronal Cre expression (Figures 4E, 4F, S4A, and S4B). By limiting Cre expression to neuronal subsets, we observed full rescue when *dmsr-7* was expressed only in the RIC and RIM interneurons (Figures 4G and S4C) and partial rescue when *dmsr-7* was expressed only in RIC interneurons (Figures 4H and S4D). We also observed full rescue when *dmsr-7* was expressed in AVK neurons, suggesting a feedback function, and rescue in cholinergic neurons that did not overlap with RIC, RIM, or AVK (Figures 4I, 4J, S4E, and S4F). A feedback function of *flp-1/dmsr-7* on AVK can also affect locomotion in the absence of pathogen.^40^ We did not observe rescue when *dmsr-7* was expressed in a set of neurons including RIM but not RIC (*glr-1+*) or in ventral cord motor neurons (*acr-2+*) (Figures 4K, 4L, S4G, and S4H).

### Mutations in tyramine/octopamine synthesis suppress *dmsr-7* defects

**Figure 5:**
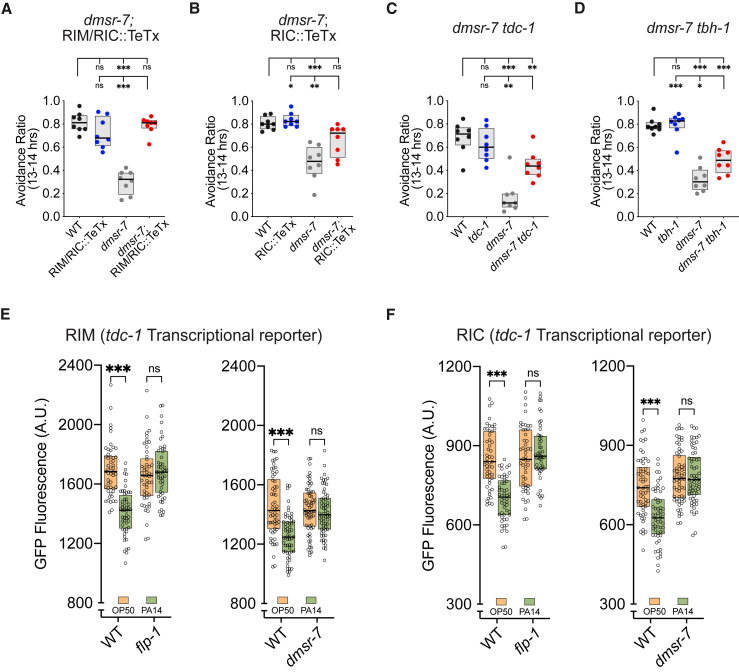
PA14 and FLP-1/DMSR-7 signaling antagonize tyramine/octopamine signaling (A and B) Expression of tetanus toxin light chain in RIM and RIC (A) or RIC alone (B) suppressed the PA14 avoidance defect in *dmsr-7* mutants. (C) A *tdc-1(n3419)* mutation that inactivates tyrosine decarboxylase suppressed the avoidance defect of *dmsr-7* mutants. (D) A mutation in *tbh-1* (tyramine beta-hydroxylase) partially suppressed the avoidance defect of *dmsr-7* mutants. (E and F) PA14 exposure decreased expression of a *tdc-1*p::GFP reporter in RIM (E) and RIC (F) neurons in WT but not in *flp-1* and *dmsr-7* mutants. Complete 20 h time courses for (A)–(D) are shown in Figure S5. For (A)–(D), *n* = 8 assays for all groups. For (E) and (F): left, WT in OP50, *n* = 50; WT in PA14, *n* = 52; *flp-1* in OP50, *n* = 50; *flp-1* in PA14, *n* = 49; right, WT in OP50, *n* = 58; WT in PA14, *n* = 57; *dmsr-7* in OP50, *n* = 62; *dmsr-7* in PA14, *n* = 62. For all experiments, boxplot center lines denote median; box ranges denote 25th–75th percentiles. ^∗^p < 0.05, ^∗∗^p < 0.01, and ^∗∗∗^p < 0.001, ns, not significant: (A–D) one-way ANOVA with Tukey’s post hoc test and (E and F) two-way ANOVA with Tukey’s post hoc test.

To expand our understanding of this circuit, we focused on the RIC and RIM neurons. DMSR-7 is related to the *Drosophila* myosuppressin receptor 1, which signals through an adenylate-cyclase-inhibiting GPCR pathway.^41^ Similarly, DMSR-7 expressed in heterologous cells is Gi coupled and inhibits cAMP signaling.^39^ We reasoned that DMSR-7 might promote avoidance by inhibiting RIC and RIM during exposure to PA14. In agreement with this hypothesis, expressing the tetanus toxin light chain to block neurotransmitter release from RIC and RIM or from RIC alone restored PA14 avoidance to *dmsr-7* mutants (Figures 5A, 5B, S5A, and S5B). Expressing tetanus toxin in these neurons in wild-type animals had minimal effects on avoidance. These results suggest that synaptic release from RIC and RIM can inhibit PA14 avoidance, and FLP-1 signaling through DMSR-7 opposes this function.

The RIC and RIM interneurons produce the neurotransmitters glutamate and tyramine as well as several neuropeptides, and RIC also produces octopamine.^25^^,^^42^^,^^43^ A loss-of-function mutation in the tyrosine decarboxylase *tdc-1* that eliminates both tyramine and octopamine restored PA14 avoidance in *dmsr-7* mutants, and a *tbh-1* (tyramine beta-hydroxylase 1) mutation that selectively decreases octopamine partially restored avoidance (Figures 5C, 5D, S5C, and S5D). As single mutants, both *tdc-1* and *tbh-1* exhibited normal PA14 avoidance. These results suggest that inhibition of tyraminergic/octopaminergic signaling after PA14 exposure contributes to avoidance behavior.

A transcriptional reporter in which the *tdc-1* promoter drove GFP expression was moderately downregulated in both RIC and RIM neurons after PA14 exposure (Figures 5E and 5F). PA14 did not downregulate *tdc-1*p::GFP in *dmsr-7* or *flp-1* mutant backgrounds, suggesting that FLP-1 signaling to DMSR-7 regulates *tdc-1* transcription in RIC and RIM (Figures 5E and 5F). Interestingly, whole-animal metabolite analysis showed changes in the levels of tyramine, octopamine, and their metabolites in animals exposed to PA14 for 12 h compared to animals maintained on *E. coli* (Figures S5E and S5F). Tyramine levels were substantially reduced and octopamine levels increased. While intriguing, these changes were also observed in *flp-1* mutants*,* suggesting that PA14 regulates tyramine/octopamine signaling through multiple mechanisms and not just through *flp-1/dmsr-7* signaling. This is not surprising, as exposure to PA14 is a substantial insult to the animal, and tyramine and octopamine are also made by non-neuronal gonadal cells that are likely to contribute to whole-body metabolic changes.^42^^,^^44^

### FLP-1/DMSR-7 and DAF-7/TGF-β regulate pathogen avoidance through partly overlapping mechanisms

Previous work demonstrated that the RIC and RIM neurons contribute to PA14 avoidance mediated by the TGF-β-related cytokine DAF-7.^22^ When ASJ chemosensory neurons detect the PA14 metabolites PCN and pyochelin, they induce transcription of *daf-7*, which acts on the DAF-1/DAF-4 TGF-β receptor in RIC and RIM to inhibit the co-SMAD transcription factor DAF-3.^22^ Because *daf-7* and *dmsr-7* converge at the RIC and RIM neurons, we examined their relationship with each other and other regulators of pathogen avoidance.

**Figure 6:**
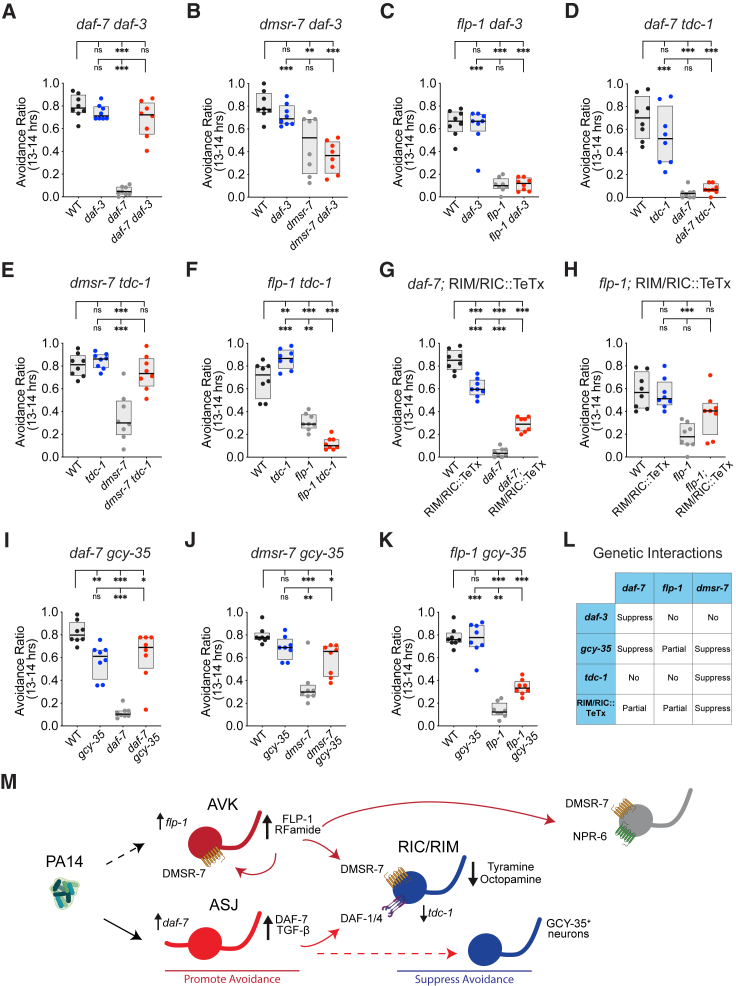
DAF-7 and FLP-1 converge on RIM/RIC interneurons but act through distinct pathways (A–C) A *daf-3* mutation suppressed the PA14 avoidance defect of *daf-7* (A) but not *dmsr-7* (B) or *flp-1* (C). (D–F) A *tdc-1(n3420)* mutation suppressed the PA14 avoidance defect of *dmsr-7* (E) but not *daf-7* (D) or *flp-1* (F). (G and H) Expression of tetanus toxin in RIM and RIC partially suppressed the PA14 avoidance defects of *daf-7* (G) and *flp-1* (H) (for full suppression of *dmsr-7,* see Figure 5B). (I–K) A *gcy-35* mutation suppressed the PA14 avoidance defect of *daf-7* (I) and *dmsr-7* (J) but only partially suppressed *flp-1* (K). (L) Summary of genetic interactions. (M) Summary: AVK neurons release FLP-1 neuropeptides during PA14 exposure to promote pathogen avoidance; FLP-1 acts on the GPCR DMSR-7 to suppress antagonistic tyramine/octopamine signaling from RIM and RIC. Other targets of FLP-1 include AVK itself (via *dmsr-7*) as well as unidentified cells that express DMSR-7 and NPR-6. PA14 metabolites increase expression of DAF-7 TGF-β in ASJ sensory neurons; DAF-7 acts through its receptors on RIM and RIC neurons to inhibit *gcy-35-*mediated oxygen avoidance.^22^ Red, neurons that promote pathogen avoidance. Blue, neurons that suppress pathogen avoidance. Complete 20 h time courses for (A)–(K) are shown in Figure S6. For assays in (D) and (I), all genotypes were grown at 15°C to prevent *daf-7(ok3125)* dauer larva formation, shifted to 20°C when they reached the L2/L3 larval stage, and tested as L4s at 21°C. For (A)–(K), *n* = 8 assays for all groups. For all experiments, boxplot center lines denote median; box ranges denote 25th–75th percentiles. ^∗^p < 0.05, ^∗∗^p < 0.01, and ^∗∗∗^p < 0.001, ns, not significant, by one-way ANOVA with Tukey’s post hoc test.

Loss-of-function mutants of *daf-7* or *daf-1* display severe defects in PA14 avoidance that are fully suppressed by *daf-3*^22^ (Figures 6A, S6A, and S6G). However, a *daf-3* mutation did not restore PA14 avoidance to *dmsr-7* mutants (Figures 6B and S6B). Conversely, while the tyrosine decarboxylase mutant *tdc-1* suppressed *dmsr-7*, it did not suppress *daf-7*^22^ (Figures 6D, 6E, S6D, and S6E). This double dissociation indicates that *daf-7* and *dmsr-7* have distinct targets even though they both act on RIM and RIC neurons.

Other mutants and manipulations had more complex genetic interactions. First, *flp-1* defects in PA14 avoidance were not suppressed by either *daf-3* or *tdc-1* (Figures 6C, 6F, S6C, and S6F). Second, the expression of tetanus toxin in RIC and RIM, which fully suppressed *dmsr-7*, only partially suppressed *flp-1* and *daf-7* (Figures 5A, 6G, 6H, S5A, S6H, and S6I). Finally, the oxygen-sensing guanylate cyclase *gcy-35*, which suppresses *daf-7*,^22^ suppressed *dmsr-7* but only partially suppressed *flp-1* (Figures 6I–6K and S6J–S6L). We conclude that *flp-1*, *dmsr-7*, and *daf-7* are antagonized by overlapping, but not identical, signaling molecules in PA14 avoidance. Among these genes, *flp-1*, which acts through multiple receptors on multiple cells, was the most robust to the antagonistic effects of tyramine/octopamine, RIM/RIC, and the oxygen-sensor *gcy-35* (Figure 6L).

## Discussion

Transitions between behaviors often engage antagonistic neuromodulators that stabilize alternative circuit states. For example, in the arcuate nucleus of the rodent hypothalamus, antagonistic and interconnected NPY/AgRP and POMC neurons release AgRP and αMSH neuropeptides to direct feeding and satiety, respectively.^45^ In *C. elegans*, antagonism between serotonin and the neuropeptide PDF-1 stabilize opposing dwelling and roaming states, modulating behavior in the timescale of minutes.^46^ Here, we show that pathogen avoidance is regulated over hours by opposing functions of FLP-1 neuropeptides released by the AVK neurons and tyramine/octopamine neuromodulators produced by RIM and RIC neurons (Figure 6M).

Through a survey of candidate genes, followed by cellular and molecular analysis, we identified FLP-1 neuropeptides and AVK neurons as key regulators of PA14 avoidance. AVK neurons specify the dynamics of PA14 avoidance behavior: reducing their activity or synaptic release delays avoidance, and enhancing their synaptic release accelerates avoidance. PA14 appears to stimulate FLP-1 release, although the indirect nature of the FLP-1 release reporter could mask more complex explanations for its regulation.

AVK-derived FLP-1 neuropeptides potently activate the GPCR DMSR-7, among other receptors.^39^ We found that DMSR-7 has a major role in pathogen avoidance and a second receptor, NPR-6, has a minor effect. *dmsr-7* can act in at least three different classes of neurons downstream of *flp-1*: RIC, RIM, and AVK. *npr-6* is not expressed in these neurons, suggesting that FLP-1 acts on other cellular targets as well.^25^ The requirements for *flp-1* in pathogen avoidance differ from those in locomotion, where *frpr-7* and *npr-6* (the latter in SMB neurons) are the most important receptors.^31^^,^^47^

Blocking neurotransmitter release from RIC and RIM neurons or mutating the biosynthetic genes for their tyramine and octopamine transmitters restores pathogen avoidance to *dmsr-7* mutants; RIC appears to have a larger effect than RIM. These results suggest that RIC and RIM antagonize pathogen avoidance and that they are inhibited by *dmsr-7.* An inhibitory role of DMSR-7 is consistent with its coupling to Gi proteins,^39^ which inhibit synaptic release and regulate CREB transcription factors.^48^^,^^49^^,^^50^ Octopamine from RIC also inhibits innate immunity to PA14,^51^ an activity that is reminiscent of the immunosuppressive effects of its vertebrate counterpart, adrenaline.^52^^,^^53^ Thus, the downregulation of RIC activity by PA14 enables both physiological and behavioral responses to pathogen.

In pathogen avoidance, RIC and RIM are also the targets of the TGF-β*-*related cytokine *daf-7*.^22^ Despite their shared function in RIC and RIM, *daf-7* and *dmsr-7* differ in their dependence on the *daf-3* transcription factor and tyramine/octopamine transmitters, respectively. *flp-1* does not require either *daf-3* or tyramine/octopamine, consistent with its action on a larger set of receptors and cells (Figure 6M).

AVK is the most highly connected neuron in the *C. elegans* neuropeptide connectome; it expresses the most neuropeptides and neuropeptide receptors and broadcasts this information to the most target neurons.^54^ We speculate that interoceptive detection of pathogens or tissue damage activates AVK and promotes FLP-1 release.^55^ This proposed function would complement the chemosensory detection of external PA14 metabolites by ASJ and *daf-7*.^22^ AVK’s broadcasting function may orchestrate responses to other pathogens as well; an independent study showed that *flp-1* and *dmsr-7*, as well as several other neuropeptides, affect avoidance of pathogenic *Vibrio cholerae*.^56^ At the same time, AVK is not solely a pathogen-detecting neuron because AVK and its component neuropeptides have locomotor functions in the absence of pathogens.^31^^,^^40^^,^^57^ Instead, the aggregate results support a distributed and combinatorial logic for the action of FLP-1 neuropeptides in multiple behaviors.

Systematic studies of neuropeptide-receptor interactions in *C. elegans* show that most neuropeptides act on multiple receptors, which are in turn expressed on multiple neurons.^25^^,^^39^^,^^54^ The different functions of *flp-1* in pathogen avoidance and locomotion indicate that neuropeptide release from a single neuron can act through different receptors and cells in different contexts. Moreover, neuropeptide release itself can be regulated with remarkable specificity: in locomotion, AVK-derived FLP-1 inhibits AVK release of an antagonistic neuropeptide, NLP-10.^40^ This result further highlights the combinatorial nature of neuromodulation. We attempted to preserve the natural regulatory context of *egl-3*, *flp-1*, and *dmsr-7* by expressing them in subsets of their normal expression patterns, but in some cases, even these experiments may have generated unnatural combinations of neuromodulatory signals.

Neuropeptide gene expression is increased by neuronal activity in *C. elegans* and in mammals, suggesting a general role for neuromodulatory transcription in the slow modification of circuit function.^58^^,^^59^^,^^60^^,^^61^ We found that transcriptional reporters for *flp-1* and *tdc-1* were oppositely regulated by PA14, and previous studies have shown that PA14 regulates expression of other relevant neuromodulatory genes including *daf-7**^22^* and the serotonin biosynthetic gene *tph-1*.^17^^,^^62^ The interactions between these neuromodulatory systems at the levels of gene expression, regulated secretion, neuronal activity, and metabolism are compelling topics for future study.

### Limitations of the study

The distributed nature of the pathogen avoidance circuit, and of neuromodulatory circuits more generally, means that the combination of genes and neurons identified here may not be unique. For example, FLP-1 neuropeptides can activate at least nine different GPCRs^39^ and possibly more. While our results point to DMSR-7 as one key receptor, there may be other FLP-1 receptors that are also required for pathogen avoidance. Even for the DMSR-7 and NPR-6 receptors identified here, only a subset of the neurons that regulate avoidance have been characterized, and the results point to redundancy and functional overlap between neurons. Turning to FLP-1 target neurons, the contributions of the DMSR-7-expressing RIM and RIC neurons to pathogen avoidance may not be identical to those of tyramine and octopamine. Both the neurons and the modulators are important, but RIM and RIC produce other transmitters,^25^ and tyramine and octopamine are produced by other tissues.^42^ Finally, we have not directly monitored the effects of pathogens on neuronal activity, limiting our mechanistic understanding of the neuropeptidergic circuit.

## STAR★Methods

### Key resources table

**Table:** 

REAGENT or RESOURCE	SOURCE	IDENTIFIER
**Bacterial and virus strains**		

*Pseudomonas aeruginosa* PA14	J. N. Engel	N/A
*E. coli* OP50	Brenner^63^	RRID:WB-STRAIN:WBStrain00041969

**Chemicals, peptides, and recombinant proteins**		

DifcoTM Agar, Bacteriological	BD	Cat#214510
DifcoTM Peptone	BD	Cat#211677
Sodium Chloride	Fisher Scientific	Cat#S640-500
Potassium Phosphate Dibasic	Sigma-Aldrich	Cat#P3786-100G
Potassium Phosphate Monobasic	Fisher Scientific	Cat#P285-500
Calcium Chloride Dihydrate	Sigma-Aldrich	Cat#C3881-500G
Magnesium Sulfate Anhydrous	Fisher Scientific	Cat#M65-500
Cholesterol	Sigma-Aldrich	Cat#C3045-25G
Sodium Azide	Sigma-Aldrich	Cat#S2002
5-Fluoro-2′-deoxyuridine (FudR)	Sigma-Aldrich	Cat#F0503-250MG
Histamine Dihydrochloride	Sigma-Aldrich	Cat#H7250-5G
Coelenterazine	Invitrogen	Cat#C6780
Phosphate Buffered Saline (PBS, 10X, pH 7.2)	Gibco	Cat#70013-032

**Critical commercial assays**		

Gibson Assembly Kits	New England Biolabs	Cat#E2611S
DMEM/F-12, no phenol red	Gibco	Cat#21041025
Lipofectamine LTX with Plus reagent	Invitrogen	Cat#15338100
Q5 High Fidelity 2x Master Mix	New England Biolabs	Cat#M0494S

**Deposited data**		

Avoidance ratios and quantitative imaging data	This study	Mendeley Data: https://doi.org/10.17632/yswwmx8csy.1

**Experimental models: Cell lines**		

CHO-K1 cells	PerkinElmer	ES-000-A24

**Experimental models: Organisms/strains**		

*C. elegans*: N2 (Bristol) Wild-type	This study	CX0006
*C. elegans*: VC671 *egl-3(ok979)* V	*Caenorhabditis* Genetics Center	RRID:WB-STRAIN:WBStrain00035966
*C. elegans*: MT1241 *egl-21(n611)* IV	*Caenorhabditis* Genetics Center	RRID:WB-STRAIN:WBStrain00026827
*C. elegans*: KP2018 *egl-21(n476)* IV	*Caenorhabditis* Genetics Center	RRID:WB-STRAIN:WBStrain00023630
*C. elegans*: PS8997 *flp-1(sy1599)* IV	*Caenorhabditis* Genetics Center	RRID:WB-STRAIN:WBStrain00051001
*C. elegans*: MT13113 *tdc-1(n3419)* II	*Caenorhabditis* Genetics Center	RRID:WB-STRAIN:WBStrain00027424
*C. elegans*: MT9455 *tbh-1(n3247)* X	*Caenorhabditis* Genetics Center	RRID:WB-STRAIN:WBStrain00027363
*C. elegans*: QW2 *zfIs1*[*tdc-1*p::GFP *+ lin-15 (n765ts)* rescue]	Mark Alkema	RRID:WB-STRAIN:WBStrain00044905
*C. elegans*: CB1376 *daf-3(e1376)* X	*Caenorhabditis* Genetics Center	RRID:WB-STRAIN:WBStrain00004312
*C. elegans*: CB1372 *daf-7(e1372)* III	*Caenorhabditis* Genetics Center	RRID:WB-STRAIN:WBStrain00004310
*C. elegans*: JT5464 *daf-7(e1372)* III*; daf-3(e1376)* X	James Thomas	JT5464
*C. elegans*: MT10548 *tdc-1(n3420)* II	Robert Horvitz	MT10548
*C. elegans*: ZD910 *tdc-1(n3420)* II*; daf-7(ok3125)* III	Meisel et al.^22^	ZD910
*C. elegans*: ZD634 *gcy-35(ok769)* I*; daf-7(ok3125)* III	Meisel et al.^22^	ZD634
*C. elegans*: ZX1836 *npr-6(tm1497) frpr-7(gk463846)* X	Oranth et al.^31^	ZX1836
*C. elegans*: DR40 *daf-1(m40)* IV	*Caenorhabditis* Genetics Center	RRID:WB-STRAIN:WBStrain00006167
Transgenic and mutant *C.elegans* strains	This study	Table S5

**Recombinant DNA**		

pJMS01 *egl-3*p::*egl-3*(Genomic):SL2:GFP	This study	pJMS01
pJMS02 *flp-1*(513bp)p:nCre	This study	pJMS02
pJMS03 *flp-1*(513bp)p:HisCl1	This study	pJMS03
pJMS04 *twk-47*p::*pkc-1(gf)*:SL2:GFP	This study	pJMS04
pJMS05 *flp-1*p(1.5 kb):*flp-1*(Genomic):SL2:GFP	This study	pJMS05
pJMS06 *flp-1*p(1.5 kb):GFP	This study	pJMS06
pJMS07 *dmsr-7*p::*dmsr-7*(Genomic):SL2:GFP	This study	pJMS07
pJMS08 *dmsr-7*p::*dmsr-7* Inverted Rescue	This study	pJMS08
pJMS09 *twk-47*p::nCre	This study	pJMS09
pJMS10 *flp-1*(1.5 kb)p:TeTx:SL2:GFP	This study	pJMS10

**Software and algorithms**		

MATLAB (2017a)	MathWorks	https://www.mathworks.com/products/matlab.html
ImageJ (v1.53c)	National Institutes of Health	https://imagej.nih.gov/ij/
Streampix (v7.0.2.0)	Norpix	https://www.norpix.com/products/streampix/streampix.php
Prism9 (v9.3.1 and v8.4.0)	Graphpad	https://www.graphpad.com/scientific-software/prism/
Xcalibur 4.1 QualBrowser (v4.1.31.9)	Thermo Scientific	https://www.thermofisher.com/order/catalog/product/OPTON-30965
MS Excel (v2112)	Microsoft	http://office.live.com/start/Excel.aspx
Adobe Illustrator 2022	Adobe	https://www.adobe.com/creativecloud.html
Custom MATLAB Count Lawn Leaving Code	This study	Zenodo: https://doi.org/10.5281/zenodo.10723701

**Other**		

AxioObserver Z1	Zeiss	N/A
Inverted Axio Observer Z1 LSM 780	Zeiss	N/A
PL-D7715 Camera	PixeLink	Cat#PL-D7715
MicroBeta LumiJet Luminometer	PerkinElmer	N/A

### Resource availability

#### Lead contact

Further information and requests for resources and reagents should be directed to and will be fulfilled by the lead contact, Cornelia I. Bargmann (cori@rockefeller.edu).

#### Materials availability

All unique reagents generated in this study are available from the lead contact without restriction. See Table S5 for a detailed list of the strains generated in this study.

#### Data and code availability

• All Avoidance Ratio data and quantitative microscopy data have been deposited at Mendeley Data and are publicly available as of the date of publication. DOIs are listed in the key resources table.
• All original code has been deposited at Zenodo and is publicly available as of the date of publication. DOIs are listed in the key resources table.
• Any additional information required to reanalyze the data reported in this paper is available from the lead contact upon request.

### Experimental model and study participant details

#### Nematode culture

*C. elegans* strains were maintained at room temperature (20°C–22°C) on nematode growth medium (NGM) plates seeded with *E. coli* OP50 as food source, as previously described by Brenner, 1974.^63^ Wild-type animals were CX0006, a descendant of *C. elegans* Bristol N2. Mutant strains used for the candidate gene screen are described in Table S1. Strains and controls were raised together under identical conditions.

#### PA14 cultivation

*P. aeruginosa* PA14 was a gift from J. N. Engel, UCSF. To obtain single colonies of PA14, 10 cm Luria broth (LB) agar plates were warmed up to 37°C, streaked with a PA14 frozen glycerol stock, and incubated at 37°C for 18–20 h. The streaked plate was stored at 4°C and used within a week. Single colonies of PA14 were cultured in 3 mL of LB at 25°C for ∼20 h with agitation (250 rpm). The optical density of the liquid culture was measured at 600 nm (OD_600_). To reduce variability within assays, only liquid cultures in the OD_600_ range 4–4.5 were used.

NGM plates for PA14 avoidance assays were prepared by mixing 3 g of NaCl, 2.5 g of peptone, 22 g of agar and 1 mL of cholesterol (5 mg/mL in ethanol) in 1 L; autoclaved for 45 min, and cooled to 55°C before adding 1 mL 1M CaCl_2_, 1 mL 1M MgSO_4_, and 25 mL 1M KPO_4_. 10 mL of agar was dispensed into 6 cm plates, which were kept at room temperature for 2 days and stored in an air-tight container at room temperature for up to two weeks. Standard and full-lawn PA14 killing plates were prepared as described with the addition of 50 μg/mL 5-fluorodeoxyuridine (FudR) to the NGM to sterilize the animals.

Plates for PA14 avoidance assays (standard plates) were prepared by seeding 10 μL of PA14 liquid culture in the center of 6 cm NGM plates, generating a circular bacterial lawn. The PA14 liquid culture was mixed thoroughly by pipetting up and down until it appeared green. Prior to the seeding step, the NGM plates were air-dried without a lid for 20 min in a laminar flow hood. For full-lawn PA14 plates, 200 μL of PA14 liquid culture was spread to fully cover 6 cm NGM plates and excess liquid was removed with a pipette. Seeded plates were placed in a 25°C incubator for 24 h and then moved to room temperature for 30–60 min before transferring animals. We observed variation on PA14 lawn morphology and only examined behaviors when the bacterial lawn had a uniform center, a thick peripheral ring (“coffee stain”), and no spreading beyond the ring boundary.

### Method details

#### PA14 avoidance assay

Avoidance assays were initiated by transferring 13–17 animals in the L4 larval stage from their OP50 cultivation plate to a PA14 lawn or a control OP50 lawn. Pathogen avoidance is sensitive to the number of animals in an assay, but stable within this range.^64^ Animals were transferred first to an unseeded NGM plate, allowed to clean themselves from the bacteria for 2 min, then transferred to the assay plate next to the bacterial lawn. Assay plates were placed in a temperature-controlled behavioral setup (21°C) inside a behavioral room (21°C). The L4 to adult molt occurred approximately 4 h after transfer, implying that avoidance behavior was detected in young adult animals. Four 15 MP cameras (PL-D7715, Pixelink) were used to record up to 4 assay plates per camera at a rate of 1 frame per minute for 20 h. After 20 h, the number of animals on each plate was counted and plates where 3 or more animals were missing were discarded. Custom made MATLAB code (https://doi.org/10.5281/zenodo.10723701) was used to quantify the Avoidance Ratio: (number of animals outside the bacterial lawn)/(number of animals at the end of the assay) every 30 min.

Although reproducible at a qualitative level, the dynamics of the avoidance assays showed quantitative variability from month to month at intermediate timepoints. For example, the time at which 50% of wild-type animals avoided the lawn varied between 7 and 12 h across blocks of experiments. To control for this issue, mutant and transgenic strains were compared to matched controls collected at the same time in all figures and statistical analyses. The choice of 7–8 h and 13–14 h as intermediate timepoints spanned the 50% avoidance timepoint for wild-type and yielded robust comparisons across genotypes within and between experiments.

#### PA14 killing assay

For standard PA14 killing assays, 17–20 animals of each genotype were assayed in triplicate following transfer to a PA14 lawn as described above. For full-lawn PA14 killing assays, 45–60 animals of each genotype were assayed in duplicate. Plates were placed on a 25°C incubator and survivors scored at least twice a day.

#### *egl-3* floxed allele and cell-specific inactivation

CRISPR/Cas9 was used for replacing the *egl-3* locus with a donor template by homology directed repair. The donor template contained, in the following order, a left homology arm, a *LoxP* site 70 base pairs upstream of the *egl-3* start codon, the endogenous *egl-3* gene and its 3′ UTR, a *loxP* site 852 base pairs downstream of the *egl-3* stop codon, mCherry sequence and the *tbb-2* 3′ UTR, the *rps-0* promoter to drive expression of the hygromycin resistance (hygR) gene and the *unc-54* 3′UTR, and a right homology arm. The link to the plasmid map containing the repair template can be found in the key resources table.

Cre expression was accomplished using cell-selective transgenes. The expression patterns of *flp-*1p(1.5 kb):Cre (*n* = 15), *flp-1*p(513 bp):Cre (*n* = 26), and *twk-47*p::Cre (*n* = 19) were characterized using a pan-neuronal Cre-reporter strain (*rimb-1*p::*loxP*::NLS:mCherry:*loxP*::NLS::GFP), where Cre expression in a given neuron at any time in development exchanges NLS-mCherry expression for NLS-GFP. We found that *flp-1*p(1.5 kb):Cre, *flp-1*p(513 bp):Cre, and *twk-47*p::Cre were expressed only in AVK in 8/15, 24/26, and 19/19 animals, respectively. The SMBD and SMDD motor neurons were inconsistently labeled by *flp-1*p(1.5 kb):Cre and *flp-1*p(513 bp):Cre. GFP reporters made with *flp*-*1*p(1.5 kb) or *flp-1*p(513 bp) were only expressed in AVK in larval and adult stages as shown in Figures 3C and S2E, and also shown by others.^31^

In the *egl-3* floxed strain, inactivation of *egl-3* in AVK using *flp-1*p(513 bp):Cre and *twk-47*p::Cre was confirmed by detection of the mCherry reporter.

#### Generation of transgenic strains

Transgenic strains, including rescue strains and Cre-expressing strains, were generated following standard microinjection protocols.^65^^,^^66^ A mix of genomic DNA or cDNA clones, a co-injection marker (*elt-2*p::nls-GFP, *myo-3*p::mCherry and/or *myo-2*p::mCherry), and empty pSM vector was injected into adult hermaphrodites. Two to four independent transgenic strains were isolated and characterized to account for variability in transgene expression.

#### CRISPR/Cas9-generated mutant strains

Putative null mutants were generated using CRISPR/Cas9 to insert a stop knock-in (STOP-IN) cassette that creates frameshifts and stop codons in all reading frames of the target gene.^67^ Below are the sequences of the insertions with 20 bp flanks, shown on (+) strand (insertions are underlined and the sequence containing the stop codons in bold).

*dmsr-1(ky1053)* mutation:

GTATATAATTTCTACCATCCGGGAAGTTTGTCCAGAGCAGAGG**TGACTAAGTGATAA**GCTAGCTATACATGCCTACTTATCAA

*dmsr-5(ky1059)* mutation:

GCAGGGTCTACACAGTATTGGGGAAGTTTGTCCAGAGCAGAGG**TGACTAAGTGATAA**GCTAGCCATCGGTATTTATGTCTTTT

*dmsr-6(ky1065)* mutation:

TTCTTGTCATTCATCCTATGGGGAAGTTTGTCCAGAGCAGAGG**TGACTAAGTGATAA**GCTAGCTGTTCTTGGATTAGCTGCCA

*dmsr-7(ky1067)* mutation:

TATCTTCACCAATTTTGTGCGGGAAGTTTGTCCAGAGCAGAGGTGAGGGAAGTTTGTCCAGAGCAGAGG**TGACTAAGTGATAA**GCTAGCATGTGGCAGTTTTATCGAGA.

#### Intersectional Cre/lox rescue

Cell-specific rescue transgenes were generated with an intersectional strategy using the Cre-Lox system to restrict *dmsr-7* expression to a subset of its endogenous expression pattern. Gibson Assembly (New England Biolabs) was used to subclone fragments of the pSM-inv[SL2-GFP] vector^46^ to generate an inactive, inverted *dmsr-7* construct (Figure 4D). First, a genomic fragment of *dmsr-7* including 5033 bp of the upstream promoter region and 815 bp of the open reading frame was cloned upstream of the first Lox2272 and *LoxP* sites. Then, the remaining *dmsr-7* fragment was cloned in an inverted orientation between the inverted SL2-GFP and the second Lox2272 and inverted *LoxP*.

Transgenic *dmsr-7(ky1067)* mutants expressing the inverted *dmsr-7* construct were injected with plasmids expressing Cre under cell-specific promoters. In these transgenic lines, the functional *dmsr-7* genomic fragment is restored only in the cells that express both Cre and the inverted *dmsr-7* construct. In all cases, restoration of the *dmsr-7* rescue fragment was confirmed by observation of GFP expression in the expected neurons, and not in other cells.

#### Histamine silencing experiments

A stock solution of 1 M histamine-dihydrochloride (Sigma-Aldrich) in deionized water was filtered and stored at −20°C. NGM-Histamine plates were prepared by adding 1 M histamine to NGM agar to a final concentration of 10 mM. Plates were left at room temperature for a day and stored at 4°C for up to a month. All animals expressing HisCl1 were assayed in plates with and without histamine alongside the corresponding parental strain that did not express the transgene.

#### GPCR activation assays

GPCR activation assays were performed using CHO-K1 cells stably expressing the luminescent Ca^2+^ indicator aequorin and the promiscuous G_α16_ protein (ES-000-A24 cell line, PerkinElmer). The cells were transfected with a *dmsr-7*/pcDNA3.1 plasmid using lipofectamine LTX and Plus reagent (Invitrogen) and grown overnight at 37°C. One day post-transfection, they were supplemented with fresh culture medium and shifted to 28°C overnight. Two days post-transfection, cells were collected in bovine serum albumin (BSA) medium (DMEM/F12 without phenol red, supplemented with 0.1% BSA) at a density of 5 million cells per mL, and loaded with 5 μM coelenterazine h (Invitrogen) for 4 h at room temperature. After a 10-fold dilution, cells (25,000/well) were challenged with synthetic peptides reconstituted in DMEM/BSA medium and luminescence was measured on a MicroBeta LumiJet luminometer (PerkinElmer) for 30 s at a wavelength of 469 nm. After 30 s, 0.1% Triton X-100 was added to lyse the cells, resulting in a maximal Ca^2+^ response that was measured for 30 s. Concentration-response measurements were done in triplicate on two independent days. For each peptide concentration, a relative calcium response (%) compared to the maximum peptide-evoked response (100% activation) was calculated. Concentration-response data were then fitted in function of log[peptide]. EC_50_ values were calculated from concentration-response curves in GraphPad Prism 7 by fitting a 3-parameter concentration-response curve.

#### Imaging and fluorescence quantification

Transgenic animals expressing *flp-1*p(1.5 kb):GFP, *flp-1*p(513 bp):FLP-1(cDNA):mCherry, or *tdc-1*p::GFP were placed on full-lawn PA14 plates to eliminate variations due to avoidance behavior. Transgenic animals grown on *E. coli* OP50 plates were randomly transferred to a full-lawn PA14 plate or a control OP50 plate, and incubated for 4 or 10 h at 20°C before imaging.

Live animals were mounted on 2% agarose pads containing 5 mM sodium azide. Fluorescence was collected with a 40X objective on a Zeiss Axio Imager.Z1 Apotome microscope with a Zeiss AxioCam MRm CCD camera (Figures 3E and 3F, 3G, 5E, 5F, and S2F), or a Zeiss Inverted Axio Observer Z1 LSM 780 laser scanning confocal microscope with a 40x objective (Figures 3C and 3D). Imaging settings were constant across all experiments. Images were processed using ImageJ (NIH, Bethesda, USA) to generate a maximum-intensity Z-projection. Quantification of fluorescence levels was performed by drawing a region of interest (ROI) around the cell body and measuring mean gray scale values.

#### Sample preparation for metabolite analysis

Biochemical analysis was conducted according to a previously described protocol.^68^ Synchronized L4 hermaphrodites (wild type N2 or mutants) maintained on *E. coli* OP50 (for free neurotransmitter measurement) or *E. coli* BW25113 (for *N*-succinyl-tyramine and *N*-succinyl-octopamine analysis) were washed three times in M9 buffer to remove residual bacteria, and approximately 1000 animals were transferred onto each NGM assay plate, previously seeded with a full lawn of either *E. coli* or PA14 and grown at 25°C for one day. Animals were maintained on assay plates at 21°C for 12 h. For each sample, ∼5,000 animals (for free neurotransmitter measurement) or ∼10,000 animals (for *N*-succinyl-tyramine and *N*-succinyl-octopamine analysis) were collected and washed in PBS buffer before flash-freezing. Three or four biological replicates were grown for each condition.

For free neurotransmitter measurements, worm pellets were lyophilized to dryness, then homogenized by shaking with 2.5 mm steel balls at 1,200 rpm for 3 min in 90 s pulses, during which samples were cooled with liquid nitrogen (SPEX sample prep miniG 1600). Carbonate buffer (Na_2_CO_3_/NaHCO_3_, 100 mM, pH 12, adjusted using 10 mM NaOH) and 14.8 mM dansyl chloride solution (in acetone, prepared and stored in the dark) were freshly prepared. To each pellet sample was added 100 μL of carbonate buffer, the pH was adjusted with 1 M NaOH until the mixture reached pH 11 and 100 μL of dansyl chloride solution was added. Mixtures were vortexed vigorously for 30 s and incubated in a water bath at 55°C. After 15 min, to each tube was added 4.2 μL of 15% formic acid to adjust pH to 7, and the samples were centrifuged at 18,000g for 5 min at 4°C. Supernatants were transferred to new 1.5 mL Eppendorf tubes and lyophilized. Dried samples were resuspended in 70 μL of methanol and ethyl acetate (1:1 v/v), vortexed for 30 s and sonicated for 15 min. Supernatants were transferred to HPLC vials and analyzed on the same day by HPLC-HRMS.

For *N*-succinyl-tyramine and *N*-succinyl-octopamine analysis, samples were lyophilized for 18–24 h using a VirTis BenchTop 4K Freeze Dryer. Following the addition of 1 mL methanol, samples were sonicated for 5 min (2 s on/off pulse cycle at 90 A) using a Qsonica Q700 Ultrasonic Processor with a water bath cup horn adaptor (Qsonica 431C2). Following sonication, an additional 3 mL of methanol was added, and the extract rocked overnight at room temperature. Samples were centrifuged (3000 x g, 22°C, 5 min) and the resulting clarified supernatant transferred to a clean 8 mL glass vial which was concentrated to dryness in an SC250EXP Speedvac Concentrator coupled to an RVT5105 Refrigerated Vapor Trap (Thermo Scientific). The resulting powder was suspended in 100 μL of methanol, followed by vigorous vortex and brief sonication. This solution was transferred to a clean microfuge tube and subjected to centrifugation (20,000 x g, 22°C, 5 min) in an Eppendorf 5417R centrifuge to remove precipitate. The resulting supernatant was transferred to an HPLC vial and analyzed by HPLC–HRMS.

#### Mass spectrometric analysis

HPLC-HRMS analysis was conducted using a Thermo Fisher Scientific Vanquish Horizon ultra-HPLC (UHPLC) System coupled to a Thermo Q Exactive hybrid quadrupole-orbitrap high-resolution mass spectrometer equipped with a heated electrospray ionization (ESI) ion source, using Thermo Scientific Xcalibur software (v.4.3.73.11). For each sample, 1 μL of methanol extract or 3 μL of derivatized extract was injected and separated using a water-acetonitrile gradient on a Thermo Scientific Hypersil GOLD C18 column (150 × 2.1 mm, 1.9 μm particle size, part no. 25002–152130) and maintained at 40°C unless otherwise stated. Optima grade solvents were purchased from Fisher Scientific. Solvent A was 0.1% formic acid in water and solvent B was 0.1% formic acid in acetonitrile. A/B gradient started at 1% B for 3 min, then increased linearly to 100% B at 20 min, followed by 100% B for 5 min, then down to 1% B for 3 min. Mass spectrometer parameters: −3 kV/+3.5 kV spray voltage, 380°C capillary temperature, 40°C probe heater temperature, 60 AU sheath flow rate, 20 AU auxiliary flow rate, 2 AU sweep gas; S-lens radio frequency level 50.0, resolution 120,000 at *m/z* 200, *m/z* range 80–1,000, AGC target 3E6. Instrument was calibrated with Pierce LTQ Velos ESI^+^ and ESI^−^ calibration solutions. Peak areas were determined using Xcalibur 4.1 QualBrowser (v.4.1.31.9, Thermo Scientific) using a 5 ppm window around the *m/z* of interest or using Metaboseek software using default settings.^69^

### Quantification and statistical analysis

Statistical analyses of behavior were performed using GraphPad Prism. For the candidate gene screen, sample size was calculated to test if, at the 20-h timepoint, the mean of the wildtype control and any of up to five mutant strains tested simultaneously were equal. By using preliminary data collected with wildtype animals (mean: 0.9253, std: 0.0672, *n* = 12), we determined that all mutant strains should be tested at least 6 times in order to find significant differences at least 2 standard deviations below the mean of wildtype controls. A possible shortcoming of this calculation is the assumption that mutant strains would have a standard deviation equal to that of the wild-type group.

## Supplemental information

Document S1. Figures S1–S6 and Tables S1–S3

Table S4. Statistical analysis, related to STAR Methods

Table S5. Strains used in this study, related to key resources table

Document S2. Article plus supplemental information
