# Supplementary material for: Antagonism between neuropeptides and monoamines in a distributed circuit for pathogen avoidance
Source: Cell Rep. 2024 Apr 3;43(4):114042. doi: 10.1016/j.celrep.2024.114042 (PMC11063628; doi:10.1016/j.celrep.2024.114042)
Supplement: Document S1. Figures S1–S6 and Tables S1–S3 [file mmc1.pdf]

Cell Reports, Volume *43*

Supplemental information

Antagonism between neuropeptides and monoamines in a distributed circuit for pathogen avoidance

Javier Marquina-Solis, Likui Feng, Elke Vandewyer, Isabel Beets, Josh Hawk, Daniel A. Colón-Ramos, Jingfang Yu, Bennett W. Fox, Frank C. Schroeder, and Cornelia I. Bargmann

Figure S1

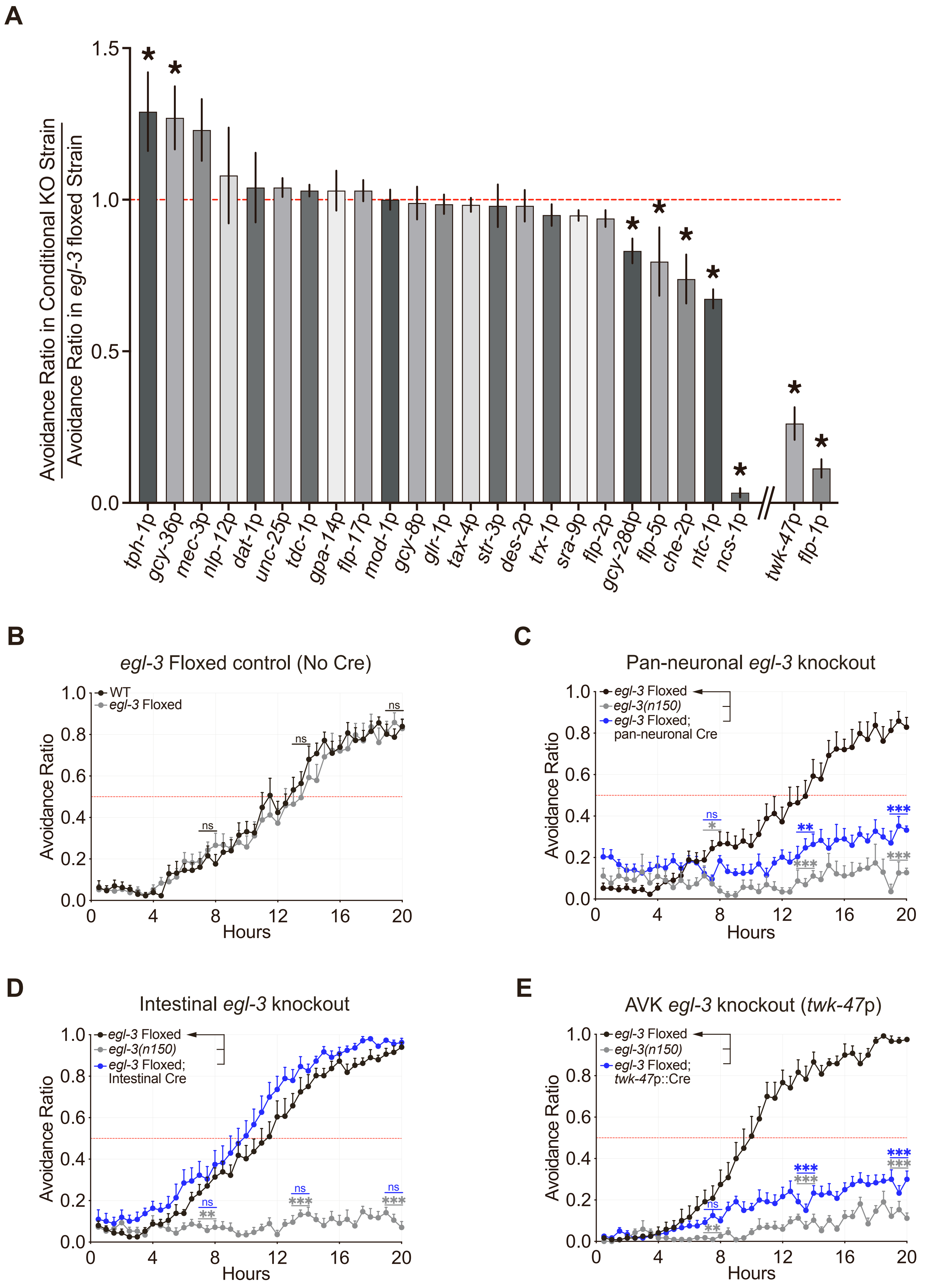

A
Avoidance Ratio in Conditional KO Strain / Avoidance Ratio in egl-3 floxed Strain
1.5
1.0
0.5
0.0
tph-1p
gcy-36p
mec-3p
nlp-12p
dat-1p
unc-25p
tdc-1p
gpa-14p
flp-17p
mod-1p
gcy-8p
glr-1p
tax-4p
str-3p
des-2p
trx-1p
sra-9p
flp-2p
gcy-28dp
flp-5p
che-2p
ntc-1p
ncs-1p
twk-47p
flp-1p
B
egl-3 Floxed control (No Cre)
WT
egl-3 Floxed
ns
Avoidance Ratio
Hours
C
Pan-neuronal egl-3 knockout
egl-3 Floxed
egl-3(n150)
egl-3 Floxed; pan-neuronal Cre
ns
*
**
***
D
Intestinal egl-3 knockout
egl-3 Floxed
egl-3(n150)
egl-3 Floxed; Intestinal Cre
ns
**
***
E
AVK egl-3 knockout (twk-47p)
egl-3 Floxed
egl-3(n150)
egl-3 Floxed; twk-47p::Cre
ns
**
***

Figure S1 | Cell-specific inactivation of *egl-3*. Related to Figure 2.

A, Cell-specific *egl-3* knockout screen for identifying neurons involved in PA14 avoidance. For each group, the Avoidance Ratio was calculated at 20 hours of exposure and normalized to *egl-3*-Floxed controls. For expression patterns, see **Table S2**.

B, *egl-3*-Floxed animals exhibit wild-type PA14 avoidance in the absence of Cre recombinase.

C, Pan-neuronal *egl-3* knockout with *rimb-1*p::Cre caused PA14 avoidance defects.

D, Intestinal *egl-3* knockout with *elt*-2p::Cre did not affect PA14 avoidance.

E, AVK *egl-3* knockout with *twk-47*p::Cre caused PA14 avoidance defects.

For **B**, n = 9 assays for all groups; for **D**, **E**, n = 8 assays for all groups; for **C**, n = 9 assays for *egl-3*-floxed, n = 4 assays for *egl-3(n150)*, n = 8 assays for *egl-3*-floxed + Pan-neuronal::Cre. For **B**, **C**, the same *egl-3*-Floxed control was used. Graphs are mean + s.e.m. $^{*}P < 0.05$, $^{**}P < 0.01$, $^{***}P < 0.001$, ns, not significant: all comparisons to *egl-3* Floxed allele (**C**-**E**) by one-way ANOVA with Dunnett's post-hoc test, (**B**) by unpaired two-tailed *t*-test.

Figure S2

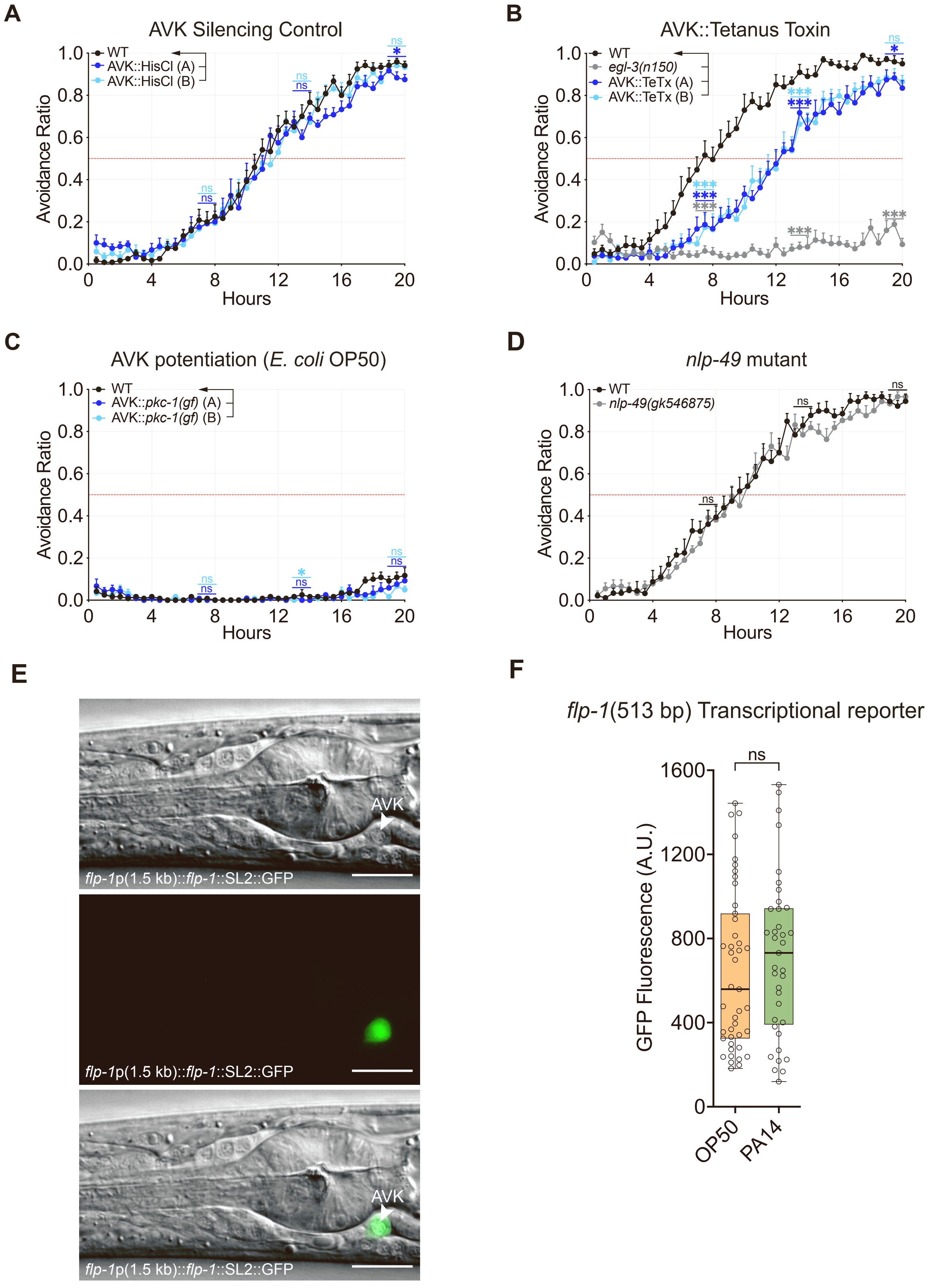
A
AVK Silencing Control
WT
AVK::HisCl (A)
AVK::HisCl (B)
Avoidance Ratio
Hours
B
AVK::Tetanus Toxin
WT
egl-3(n150)
AVK::TeTx (A)
AVK::TeTx (B)
C
AVK potentiation (E. coli OP50)
AVK::pkc-1(gf) (A)
AVK::pkc-1(gf) (B)
D
nlp-49 mutant
nlp-49(gk546875)
E
AVK
flp-1p(1.5 kb)::flp-1::SL2::GFP
F
flp-1(513 bp) Transcriptional reporter
GFP Fluorescence (A.U.)
OP50
PA14
ns

Figure S2 | Manipulations of AVK neurons, *nlp-49* mutants, and controls. Related to Figure 2 and Figure 3.

A, Animals expressing HisCl1 in the AVK neurons resemble wild-type controls in PA14 avoidance on plates without histamine.

B, Expression of the tetanus toxin light chain in AVK neurons delays PA14 avoidance.

C, Potentiation of dense core vesicle release by expression of *pkc-1(gf)* in the AVK neurons does not induce avoidance of non-pathogenic *E. coli* OP50.

D, *nlp-49(gk546875)* mutants have normal PA14 avoidance.

E, Expression of *flp-1*p(1.5 kb)::*flp-1*::SL2::GFP exclusively in AVK neurons. Top panel, Nomarski image, white arrowhead denotes AVK cell body; Middle panel, Fluorescence image (GFP); Bottom panel, overlaid images. Scale bar, 10 μm.

F, The *flp-1*p(513 bp)::GFP transcriptional reporter was not significantly induced 4 hours after exposure to PA14, although there was a trend toward increased expression. Box plot center lines denote median; box ranges denote 25–75th percentiles. OP50, n=43; PA14, n=37.

For **A**, **C**, *n* = 8 assays for all groups; for **B**, *n* = 7 assays for all groups; for **D**, *n* = 6 assays for all groups. Graphs are mean + s.e.m. *P < 0.05, **P < 0.01, ***P < 0.001, ns, not significant: all comparisons to wild type (**A-C**) by one-way ANOVA with Dunnett's post-hoc test, (**D**) by unpaired two-tailed *t*-test.

Figure S3

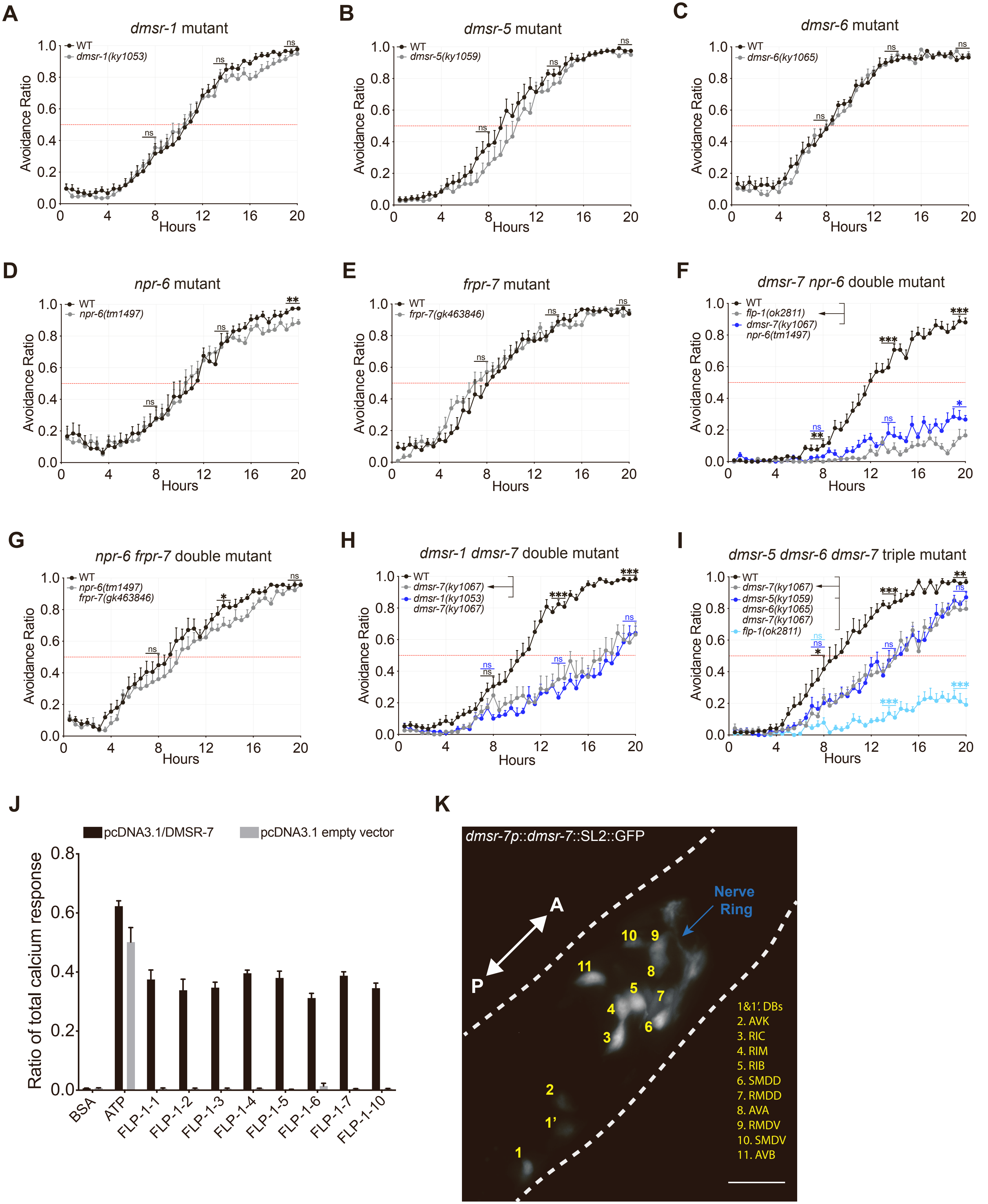
A
dmsr-1 mutant
WT
dmsr-1(ky1053)
B
dmsr-5 mutant
WT
dmsr-5(ky1059)
C
dmsr-6 mutant
WT
dmsr-6(ky1065)
D
npr-6 mutant
WT
npr-6(tm1497)
E
frpr-7 mutant
WT
frpr-7(gk463846)
F
dmsr-7 npr-6 double mutant
WT
flp-1(ok2811)
dmsr-7(ky1067) npr-6(tm1497)
G
npr-6 frpr-7 double mutant
WT
npr-6(tm1497) frpr-7(gk463846)
H
dmsr-1 dmsr-7 double mutant
WT
dmsr-7(ky1067)
dmsr-1(ky1053) dmsr-7(ky1067)
I
dmsr-5 dmsr-6 dmsr-7 triple mutant
WT
dmsr-7(ky1067)
dmsr-5(ky1059) dmsr-6(ky1065) dmsr-7(ky1067)
flp-1(ok2811)
Avoidance Ratio
Hours
J
pcDNA3.1/DMSR-7
pcDNA3.1 empty vector
Ratio of total calcium response
BSA
ATP
FLP-1-1
FLP-1-2
FLP-1-3
FLP-1-4
FLP-1-5
FLP-1-6
FLP-1-7
FLP-1-10
K
dmsr-7p::dmsr-7::SL2::GFP
A
P
Nerve Ring
1&1'. DBs
2. AVK
3. RIC
4. RIM
5. RIB
6. SMDD
7. RMDD
8. AVA
9. RMDV
10. SMDV
11. AVB

Figure S3 | FLP-1 receptor candidates, *in vitro* aequorin-based DMSR-7 activation assay controls and expression pattern of *dmsr-7* transgene. Related to Figure 4.

A-E, Normal PA14 avoidance behavior in *dmsr-1(ky1053)* (**A**), *dmsr-5(ky1059)* (**B**), *dmsr-6(ky1065)* (**C**), *npr-6(tm1497)* (**D**), and *frpr-7(gk463846)* (**E**) mutants.

F, *dmsr-7(ky1067); npr-6(tm1497)* double mutants have severe PA14 avoidance defects, resembling *flp-1(ok2811)* mutants.

G, Normal PA14 avoidance behavior in *npr-6(tm1497) frpr-7(gk463846)* double mutants.

H, I, *dmsr-7*-like PA14 avoidance behavior in *dmsr-1(ky1053) dmsr-7(ky1067)* double mutants (**H**), and *dmsr-6(ky1065); dmsr-5(ky1059); dmsr-7(ky1067)* triple mutants (**I**).

J, Calcium responses of CHO cells transfected with empty pcDNA3.1 or pcDNA3.1/DMSR-7 in response to BSA medium (Negative control), ATP (Positive control), or FLP-1 peptides. FLP-1 peptides did not elicit a response in the CHO cells transfected with empty pcDNA3.1.

K, Expression pattern of a genomic fragment containing the *dmsr-7* endogenous promoter and the complete *dmsr-7* coding region. GFP was expressed with *dmsr-7* in a bicistronic transcript. Scale bar, 10 µm.

For **A**, *n* = 12 assays for all groups; for **B-I**, *n* = 8 assays for all groups; for **J**, *n* = 8 assays for CHO cells transfected with pcDNA3.1/DMSR-7 on BSA and ATP groups, *n* = 18 assays for CHO cells transfected with pcDNA3.1 empty vector on BSA and ATP groups, for all other groups *n* = 4. For **A-F**, graphs are mean + s.e.m. P < 0.05, **P < 0.01, ***P < 0.001, ns, not significant: (**A-E, G)**, unpaired two-tailed *t*-test, (**F, H, I**) one-way ANOVA with Dunnett's post-hoc test, (**F**) comparisons to *flp-1(ok2811)*, (**H-I**) comparisons to *dmsr-7(ky1067)*.

Figure S4

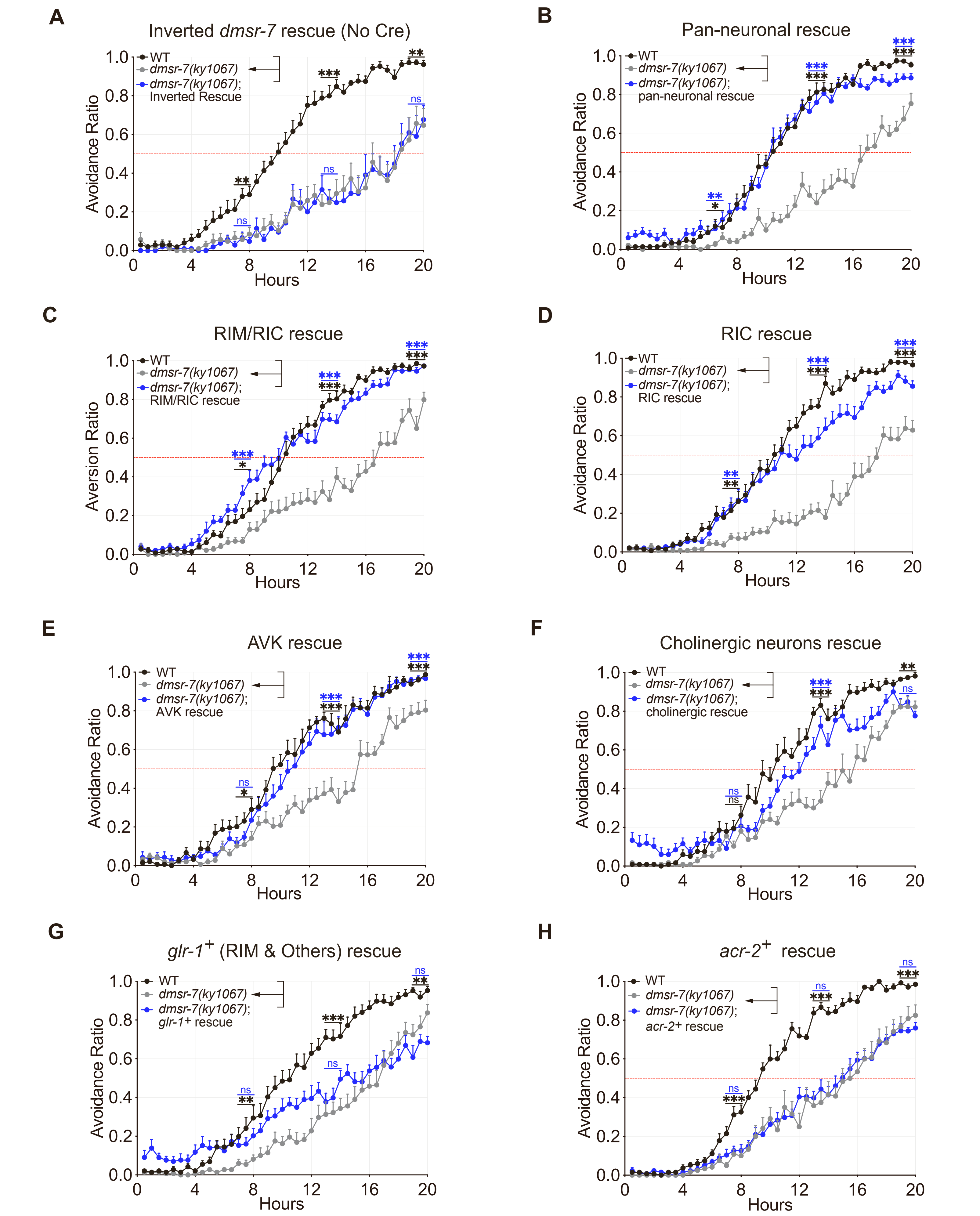

A
Inverted dmsr-7 rescue (No Cre)
WT
dmsr-7(ky1067)
dmsr-7(ky1067); Inverted Rescue
Avoidance Ratio
Hours
B
Pan-neuronal rescue
WT
dmsr-7(ky1067)
dmsr-7(ky1067); pan-neuronal rescue
Avoidance Ratio
Hours
C
RIM/RIC rescue
WT
dmsr-7(ky1067)
dmsr-7(ky1067); RIM/RIC rescue
Aversion Ratio
Hours
D
RIC rescue
WT
dmsr-7(ky1067)
dmsr-7(ky1067); RIC rescue
Avoidance Ratio
Hours
E
AVK rescue
WT
dmsr-7(ky1067)
dmsr-7(ky1067); AVK rescue
Avoidance Ratio
Hours
F
Cholinergic neurons rescue
WT
dmsr-7(ky1067)
dmsr-7(ky1067); cholinergic rescue
Avoidance Ratio
Hours
G
glr-1+ (RIM & Others) rescue
WT
dmsr-7(ky1067)
dmsr-7(ky1067); glr-1+ rescue
Avoidance Ratio
Hours
H
acr-2+ rescue
WT
dmsr-7(ky1067)
dmsr-7(ky1067); acr-2+ rescue
Avoidance Ratio
Hours

Figure S4 | Rescue of *dmsr-7* in subsets of its endogenous expression pattern. Related to Figure 4.

A, The *dmsr-7* inverted transgene did not rescue *dmsr-7(ky1067)* PA14 avoidance in the absence of Cre expression.

B-F, Rescue of *dmsr-7* avoidance behavior by Cre expression in all neurons (*rimb-1*p::Cre) (**B**), RIC and RIM interneurons (*tdc-1*p::Cre) (**C**), RIC interneurons (*tbh-1*p::Cre) (**D**), AVK interneurons (*flp-1*p(513 bp)::Cre) (**E**), and a set of interneurons and motor neurons not including RIM, RIC, or AVK (*unc-17*p::Cre, intersection in AVA, AVB, DA, DB, RIH, RMD, SMD neurons) **(F)**.

G, H, No rescue of *dmsr-7* avoidance behavior by Cre expression in RIM (*glr-1*p::Cre, intersection in AVA, RIM, RMD, SMD neurons) (**G),** or cholinergic motor neurons (*acr-2*p::Cre, intersection in DA, DB, VA, VB neurons) (**H**).

For **B-E, G**, *n* = 10 assays for all groups; for **F**, *n* = 8 assays for all groups; for **H**, *n* = 8-9 assays for all groups. Graphs are mean + s.e.m. *P < 0.05, **P < 0.01, ***P < 0.001, ns, not significant: comparisons to *dmsr-7(ky1067)* by one-way ANOVA with Dunnett's post-hoc test.

Figure S5

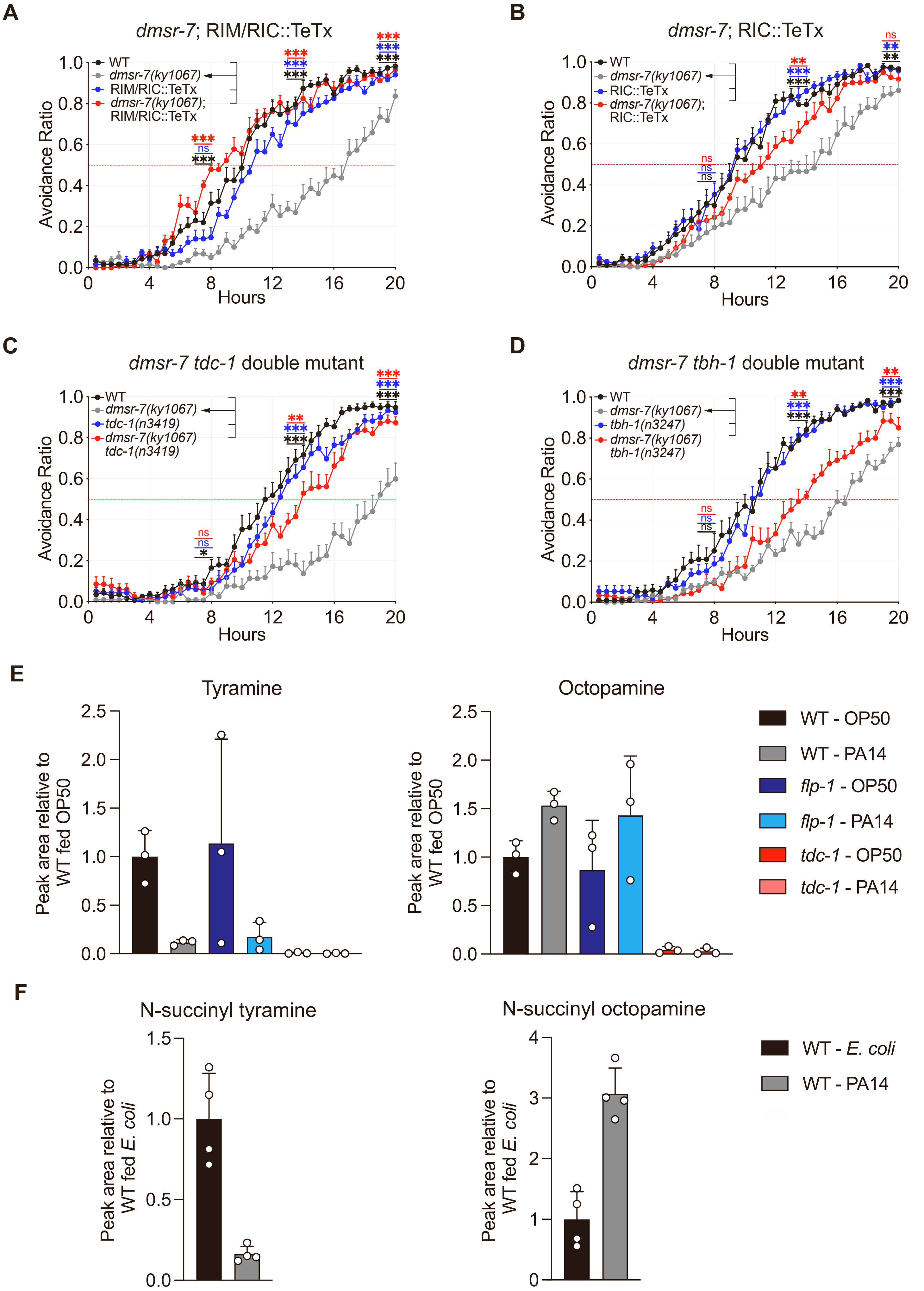
A
dmsr-7; RIM/RIC::TeTx
WT
dmsr-7(ky1067)
RIM/RIC::TeTx
dmsr-7(ky1067); RIM/RIC::TeTx
Avoidance Ratio
Hours
ns
***
B
dmsr-7; RIC::TeTx
WT
dmsr-7(ky1067)
RIC::TeTx
dmsr-7(ky1067); RIC::TeTx
Avoidance Ratio
Hours
ns
**
***
C
dmsr-7 tdc-1 double mutant
WT
dmsr-7(ky1067)
tdc-1(n3419)
dmsr-7(ky1067) tdc-1(n3419)
Avoidance Ratio
Hours
ns
*
**
***
D
dmsr-7 tbh-1 double mutant
WT
dmsr-7(ky1067)
tbh-1(n3247)
dmsr-7(ky1067) tbh-1(n3247)
Avoidance Ratio
Hours
ns
**
***
E
Tyramine
Octopamine
Peak area relative to WT fed OP50
WT - OP50
WT - PA14
flp-1 - OP50
flp-1 - PA14
tdc-1 - OP50
tdc-1 - PA14
F
N-succinyl tyramine
N-succinyl octopamine
Peak area relative to WT fed E. coli
WT - E. coli
WT - PA14

Figure S5 | DMSR-7 acts on RIM and RIC interneurons to promote PA14 avoidance. Related to Figure 5.

A, **B**, Expression of the tetanus toxin light chain in the RIM and RIC neurons (**A**) or RIC only (**B**) suppressed the *dmsr-7* PA14 avoidance defect.

C, *tdc-1(n3419),* which inactivates the tyrosine decarboxylase required for the synthesis of tyramine and octopamine, suppressed the *dmsr-7* avoidance defect.

D, *tbh-1(n3247),* which inactivates the tyramine-beta hydroxylase required for the synthesis of octopamine, partially suppressed the *dmsr-7* avoidance defect.

E, Quantification of derivatized tyramine and octopamine, as indicated, by HPLC-HRMS. WT, *flp-1* and *tdc-1* mutants were reared on *E. coli* OP50 and then transferred to *E. coli* OP50 or *Pseudomonas* PA14 for 12 hours. Tyramine levels decreased significantly in wild-type animals exposed to PA14, and octopamine levels increased. The effect of PA14 on *flp-1* did not reach significance due to variability across samples.

F, Quantification of *N*-succinyl tyramine and *N*-succinyl octopamine by HPLC-HRMS. WT animals were reared on *E. coli* BW25113 and then transferred to plates containing *E. coli* BW25113 or *Pseudomonas* PA14 for 12 hours. *N*-succinyl tyramine levels decreased significantly and *N*-succinyl octopamine levels increased significantly in PA14. *N*-succinyl conjugates represent a large fraction of the tyramine and octopamine pools in *C. elegans*[S1]. The effects of PA14 are consistent with those in (**E**).

For **A-D**, n = 8 assays for all groups; for **E**, n = 3 assays for all groups; for **F**, n = 4 assays for all groups. Graphs are mean + s.e.m. *P < 0.05, **P < 0.01, ***P < 0.001, ns, not significant: for **A-D,** all comparisons to *dmsr-7(ky1067)* by one-way ANOVA with Dunnett's post-hoc test. See Figure 5 for additional statistical comparisons.

Figure S6

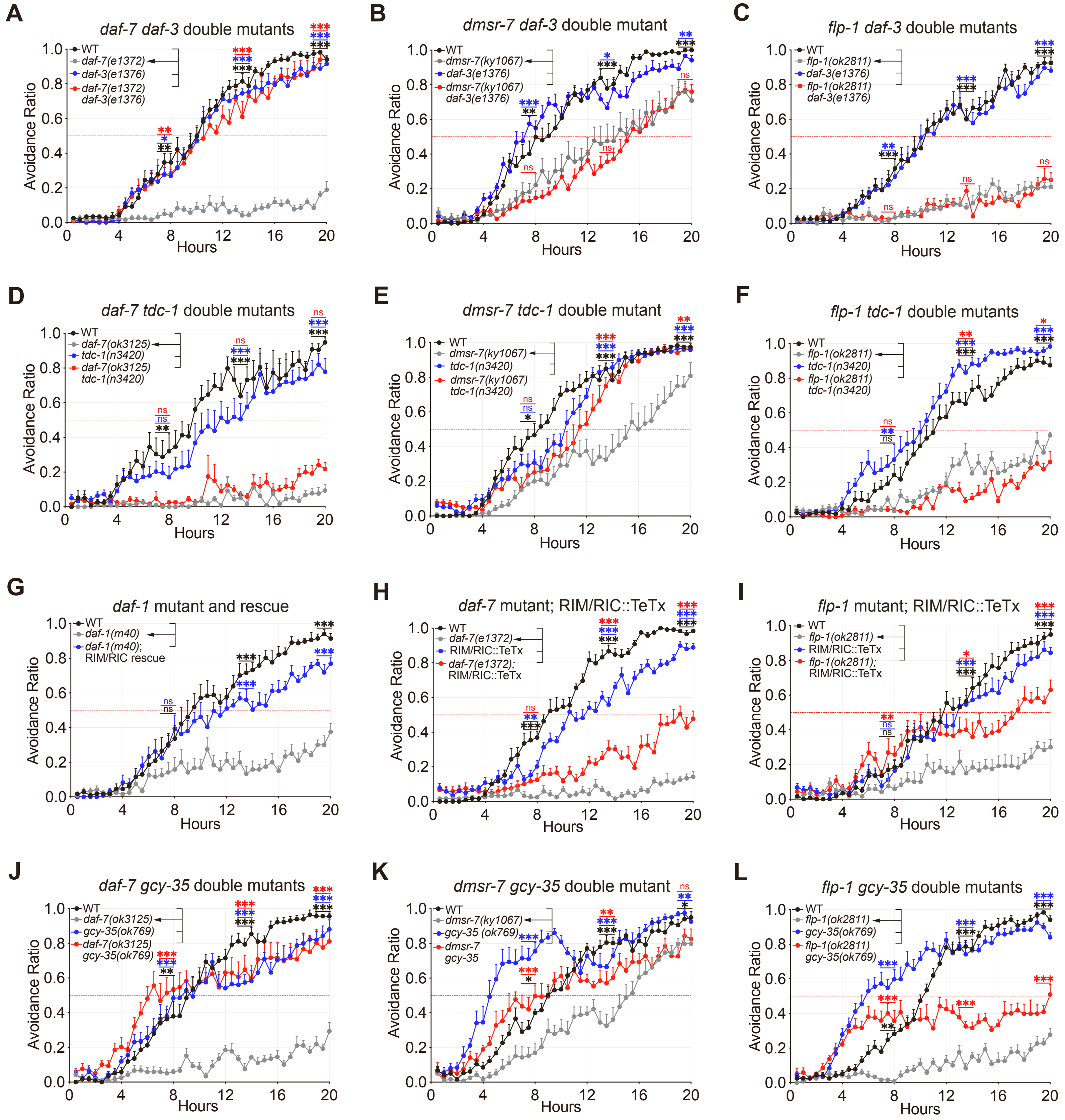
A
daf-7 daf-3 double mutants
WT
daf-7(e1372)
daf-3(e1376)
daf-7(e1372) daf-3(e1376)
B
dmsr-7 daf-3 double mutant
WT
dmsr-7(ky1067)
daf-3(e1376)
dmsr-7(ky1067) daf-3(e1376)
C
flp-1 daf-3 double mutants
WT
flp-1(ok2811)
daf-3(e1376)
flp-1(ok2811) daf-3(e1376)
D
daf-7 tdc-1 double mutants
WT
daf-7(ok3125)
tdc-1(n3420)
daf-7(ok3125) tdc-1(n3420)
E
dmsr-7 tdc-1 double mutant
WT
dmsr-7(ky1067)
tdc-1(n3420)
dmsr-7(ky1067) tdc-1(n3420)
F
flp-1 tdc-1 double mutants
WT
flp-1(ok2811)
tdc-1(n3420)
flp-1(ok2811) tdc-1(n3420)
G
daf-1 mutant and rescue
WT
daf-1(m40)
daf-1(m40); RIM/RIC rescue
H
daf-7 mutant; RIM/RIC::TeTx
WT
daf-7(e1372)
RIM/RIC::TeTx
daf-7(e1372); RIM/RIC::TeTx
I
flp-1 mutant; RIM/RIC::TeTx
WT
flp-1(ok2811)
RIM/RIC::TeTx
flp-1(ok2811); RIM/RIC::TeTx
J
daf-7 gcy-35 double mutants
WT
daf-7(ok3125)
gcy-35(ok769)
daf-7(ok3125) gcy-35(ok769)
K
dmsr-7 gcy-35 double mutant
WT
dmsr-7(ky1067)
gcy-35 (ok769)
dmsr-7 gcy-35
L
flp-1 gcy-35 double mutants
WT
flp-1(ok2811)
gcy-35(ok769)
flp-1(ok2811) gcy-35(ok769)
Avoidance Ratio
Hours

Figure S6 | *flp-1, dmsr-7,* and *daf-7* signal through different targets in RIM and RIC. Related to Figure 6.

A-**C**, *daf-3(e1376)* SMAD null mutants have normal PA14 avoidance and suppressed the *daf-7(e1372)* TGF-beta PA14 avoidance defect (**A**), but not the *dmsr-7(ky1067)* (**B**) or *flp-1(ok2811)* (**C**) avoidance defects.

D-**F**, *tdc-1(n3420)* tyrosine decarboxylase null mutants suppressed the PA14 avoidance defect of *dmsr-7(ky1067)* mutants (**E**) but not the *daf-7(ok3125)* (**D**) or *flp-1(ok2811)* (**F**) avoidance defects.

G, *daf-1(m40)* TGF-beta receptor mutants are defective in PA14 avoidance, and can be rescued by expression of *daf-1* in the RIM and RIC neurons (*tdc-1* promoter) [S2].

H, **I**, Expression of the tetanus toxin light chain in RIM and RIC neurons partially suppressed the PA14 avoidance defect of *daf-7(e1372)* (**H**) and *flp-1(ok2811)* (**I**) mutants. Note stronger suppression at the 19-20 hour timepoint than at the 13-14 hour timepoint shown in **Figure 6.**

J-**L**, *gcy-35(ok769)* guanylate cyclase mutants fully suppressed the PA14 avoidance defect of *daf-7(ok3125)* (**J**) [S2], and partially suppressed the *flp-1* avoidance defect (**L**). *gcy-35* suppressed *dmsr-7* at the 12-13 hour timepoint, but the double mutant had an intermediate phenotype at the 7-8 hour timepoint (**K**). Note accelerated avoidance of *gcy-35* single mutants in panels **K** and **L.**

In panel **D** and **J**, all genotypes were grown at 15°C to prevent dauer larva formation *in daf-7(ok3125)*, shifted to 20°C when they reached the L2/L3 larval stage, and tested as L4s at 21°C.

For **A**-**L**, n = 8 assays for all groups. Graphs are mean + s.e.m. *P < 0.05, **P < 0.01, ***P < 0.001, ns, not significant by one-way ANOVA with Dunnett's post-hoc test: (**A, H**) comparisons to *daf-7(e1372)*, (**D, J**) comparisons to *daf-7(ok3125),* (**B, E, K**) comparisons to *dmsr-7(ky1067)*, (**C**, **F, I, L**) comparisons to *flp-1(ok2811)*, and (**G**) comparisons to *daf-1(m40)*.

Strain	Gene (allele)	Backcrossed	Normalized Avoidance Ratio
CX13214	*inx-16(tm1589)*	3x	1.50 ± 0.28
RB1792	*inx-7(ok2319)*	0x	1.49 ± 0.33
RB1396	*nlp-20(ok1591)*	0x	1.31 ± 0.30
CX12726	*inx-9(ok1502)*	4x	1.28 ± 0.20
CX14394	*npr-5(ok1583)*	5x	1.26 ± 0.16
MT1222	*egl-6(n592)*	1x	1.25 ± 0.16
MT9455	*tbh-1(n3247)*	8x	1.22 ± 0.14
CX15961	*ckr-2(tm2082)*	4x	1.19 ± 0.20
MT1083	*egl-8(n488)*	1x	1.18 ± 0.11
CX13779	*nlp-5(tm2125)*	3x	1.16 ± 0.14
CX13325	*inx-2(ok376)*	4x	1.13 ± 0.10
IG685	*tir-1(tm3036)*	2x	1.12 ± 0.23
CX1030	*npr-9(ky1030)*	2x	1.12 ± 0.25
DA464	*eat-5(ad464)*	0x	1.12 ± 0.17
CX5	*eat-4(ky5)*	0x	1.09 ± 0.15
JT513	*nrf-5(sa513)*	2x	1.09 ± 0.09
RB911	*fshr-1(ok778)*	0x	1.09 ± 0.13
CX14102	*ntr-1(tm2765)*	6x	1.09 ± 0.22
MT13113	*tdc-1(n3419)*	11x	1.06 ± 0.12
RB2108	*inx-11(ok2783)*	0x	1.06 ± 0.11
VC3056	*zip-2(ok3730)*	0x	1.06 ± 0.09
CX16990	*npr-35(ok3258)*	3x	1.06 ± 0.14
CX15563	*ador-1(gk744003)*	3x	1.05 ± 0.08
VS8	*dhs-28(hj8)*	6x	1.05 ± 0.08
RB799	*npr-11(ok594)*	0x	1.04 ± 0.18
CX6448	*gcy-35(ok769)*	6x	1.03 ± 0.09
ZG31	*hif-1(ia4)*	0x	1.03 ± 0.12
RB1372	*nlp-18(ok1557)*	0x	1.03 ± 0.05
WM53	*alg-2(ok304)*	0x	1.02 ± 0.05
JT5244	*aex-4(sa22)*	3x	1.01 ± 0.04
CX16402	*npr-14(ok2375)*	4x	1.01 ± 0.05
VC8	*jnk-1(gk7)*	0x	1.01 ± 0.03
RB1341	*nlp-1(ok1470)*	0x	1.00 ± 0.05
FK171	*mek-1(ks54)*	2x	1.00 ± 0.04
IG10	*tol-1(nr2033)*	2x	0.99 ± 0.04
CX14295	*pdfr-1(ok3425)*	5x	0.96 ± 0.23

CX13851	*unc-25(e156)*	2x	0.96 ± 0.14
KB3	*kgb-1(um3)*	6x	0.95 ± 0.05
KU2	*jkk-1(km2)*	10x	0.94 ± 0.03
JT9	*aex-1(sa9)*	0x	0.93 ± 0.04
CX16403	*seb-3(gk382193)*	6x	0.90 ± 0.11
VC1063	*nlp-15(ok1512)*	0x	0.90 ± 0.04
RB1908	*mlk-1(ok2471)*	0x	0.88 ± 0.30
CX13890	*npr-4(tm1782)*	4x	0.88 ± 0.11
KU25	*pmk-1(km25)*	6x	0.85 ± 0.15
VS18	*maoc-1(hj13)*	4x	0.80 ± 0.09
NU3	*dbl-1(nk3)*	10x	0.77 ± 0.10
JN379	*nep-2(pe379)*	5x	0.76 ± 0.12*
VC1785	*acox-1(ok2257)*	0x	0.70 ± 0.13
KU4	*sek-1(km4)*	10x	0.67 ± 0.07*
CX13846	*daf-22(ok693)*	3x	0.61 ± 0.13
XM1011	*inx-22(tm1661)*	8x	0.55 ± 0.15*
KP2018	*egl-21(n476)*	5x	0.47 ± 0.05*
CX17804	*alg-1(gk214)*	6x	0.40 ± 0.11*
CX17805	*nep-17(ok3251)*	6x	0.40 ± 0.09*
CX7155	*ins-1(nr2091)*	0x	0.38 ± 0.15*
AU3	*nsy-1(ag3)*	3x	0.35 ± 0.10*
JT73	*itr-1(sa73)*	2x	0.33 ± 0.12*
CB1482	*sma-6(e1482)*	0x	0.30 ± 0.10*
CB1033	*che-2(e1033)*	0x	0.21 ± 0.11*
MT15434	*tph-1(mg280)*	?	0.20 ± 0.08*
CX9191	*egl-3(n150)*	?	0.15 ± 0.06*
CB1370	*daf-2(e1370)*	0x	0.14 ± 0.08*
CB1124	*che-3(e1124)*	0x	0.10 ± 0.03*
CX13078	*tax-4(p678)*	5x	0.05 ± 0.02*
CB1372	*daf-7(e1372)*	0x	0.04 ± 0.03*
CX4148	*npr-1(ky13)*	5x	0.00*

Table S1 | Candidate gene screen for PA14 avoidance. Related to Figure 1. Normalized Avoidance Ratio represents (Avoidance Ratio of the mutant strain)/(Avoidance Ratio of wild-type controls conducted in parallel) at 20 hours of exposure to PA14. Note that only one mutant allele was tested per gene, and therefore some phenotypes may not be caused by the gene that is named. Data shown is mean ± s.e.m. For all mutants *n* = 6 assays. *P < 0.05 by one-way ANOVA with Dunnett's post-hoc test; comparisons to respective wild-type controls.

Strain name	Promoter driving Cre	Normalized Avoidance Ratio	Neurons targeted
CX17840	*tph-1*p	1.29 ± 0.13*	ADF, NSM, HSN
CX17838	*gcy-36*p	1.27 ± 0.10*	URX, PQR, AQR
CX17836	*mec-3*p	1.23 ± 0.10	ALM, AVM, FLP, PLM, PVD, PVM
CX17852	*nlp-12*p	1.08 ± 0.16	DVA
CX17830	*dat-1*p	1.04 ± 0.12	ADE, CEPD, CEPV, PDE, OLL
CX17858	*unc-25*p	1.04 ± 0.03	RIS, VDs, DDs, AVL, DVB, RME, RIB
CX17844	*tdc-1*p	1.03 ± 0.02	RIC, RIM
CX17828	*gpa-14*p	1.03 ± 0.07	ADE, ALA, ASH, ASI, ASJ, ASK, AVA, CAN, DVA, PHA, PHB, PVQ, RIA
CX17864	*flp-17*p	1.03 ± 0.04	BAG, ASI
CX17834	*mod-1*p	1.00 ± 0.03	AIA, AIB, AIY, AIZ, RIC, RID, RIM, RME, DDs, VDs
CX17854	*gcy-8*p	0.99 ± 0.05	AFD
CX17832	*glr-1*p	0.99 ± 0.03	AIB, AVA, AVB, AVD, AVE, AVG, AVJ, DVC, PVC, PVQ, RIG, RIM, RIS, RMD, RMDD, RMDV, RME, SMDD, SMDV, URY
CX17811	*tax-4*p	0.98 ± 0.02	ASJ, AWB, ASI, URX, ASG, AWC, ASK, BAG, ASE, AFD
CX17846	*str-3*p	0.98 ± 0.07	ASI
CX17924	*des-2*p	0.98 ± 0.05	RID, others
CX17842	*trx-1*p	0.95 ± 0.04	ASJ
CX17823	*sra-9*p	0.95 ± 0.02	ASK
CX17926	*flp-2*p	0.94 ± 0.03	RID, others
CX17825	*gcy-28d*p	0.83 ± 0.04*	AIA, ASI, AVF
CX17860	*flp-5*p	0.80 ± 0.11*	RMG, ASE, PVT, I4, M4 neurons
CX17819	*che-2*p	0.74 ± 0.08*	ASE, ADE, ADF, ADL, AFD, AQR, ASG, ASH, ASI, ASJ, ASK, AWA, AWB, AWC, BAG, CEP, FLP, IL1, IL2, OLL, OLQ, PDE, PHA, PHB, PQR
CX17850	*ntc-1*p	0.67 ± 0.03*	AFD, AVK, DVA
CX18257	*twk-47*p	0.26 ± 0.05*	AVK
CX18236	*flp-1*(513 bp)p	0.11 ± 0.03*	AVK
CX17862	*ncs-1*p	0.03 ± 0.01*	ADL, AFD, AVK, ASE, ASG, ASI, AVE, AWB, AWC, BAG, RMG, 1 additional cell anterior to nerve ring, 1 tail neuron

Table S2 | Cell-directed neuropeptide screen of neurons for PA14 avoidance. Related to Figure 2 and Figure S1.

Normalized Avoidance Ratio represents (Avoidance Ratio of the conditional knockout strain)/ (Avoidance Ratio of *egl-3* floxed controls conducted in parallel) at 20 hours of exposure to PA14. Reported expression patterns of relevant promoter fragments were drawn from the literature. Data shown is mean ± s.e.m. For all conditional knockout strains, n = 6 or 8 assays. *P < 0.05 by one-way ANOVA with Dunnett's post-hoc test or by unpaired two-tailed t-test; comparisons to matched *egl-3* floxed controls.

Peptide	Sequence	log EC50(M)	95% Confidence Interval	EC50 (M)
FLP-1-4*	SDPNFLRFamide	-10.28	-10.54 to -10.03	5.23×10^{-11}
FLP-1-7*	AGSDPNFLRFamide	-10.00	-10.25 to -9.75	1.00×10^{-10}
FLP-1-5*	AAADPNFLRFamide	-9.94	-10.25 to -9.64	1.14×10^{-10}
FLP-1-2*	SQPNFLRFamide	-9.88	-10.19 to -9.57	1.31×10^{-10}
FLP-1-10*	PNFLRFamide	-9.60	-9.84 to -9.35	2.54×10^{-10}
FLP-1-1*	SADPNFLRFamide	-9.30	-9.63 to -8.97	4.97×10^{-10}
FLP-1-3*	ASGDPNFLRFamide	-9.24	-9.50 to -8.99	5.73×10^{-10}
FLP-1-6*	KPNFLRFamide	-8.68	-8.86 to -8.50	2.10×10^{-9}
FLP-1-8^	KPNFMRYamide	-6.34	-6.42 to -6.26	1.51×10^{6}
FLP-1-9^	PNFMRYamide	-5.82	-5.89 to -5.75	4.61×10^{-7}

Table S3 | Interactions of DMSR-7 with FLP-1 neuropeptides in CHO-K1 cells. Related to Figure 4 and Figure S3.

* = FLP-1 RFamide peptide, ^ = FLP-1 RYamide peptide

SUPPLEMENTAL REFERENCES

[S1] Artyukhin, A.B., Yim, J.J., Srinivasan, J., Izrayelit, Y., Bose, N., von Reuss, S.H., Jo, Y., Jordan, J.M., Baugh, L.R., Cheong, M., et al. (2013). Succinylated octopamine ascarosides and a new pathway of biogenic amine metabolism in *Caenorhabditis elegans*. J Biol Chem *288*, 18778-18783. 10.1074/jbc.C113.477000.

[S2] Meisel, J.D., Panda, O., Mahanti, P., Schroeder, F.C., and Kim, D.H. (2014). Chemosensation of bacterial secondary metabolites modulates neuroendocrine signaling and behavior of *C. elegans*. Cell *159*, 267-280. 10.1016/j.cell.2014.09.011.
